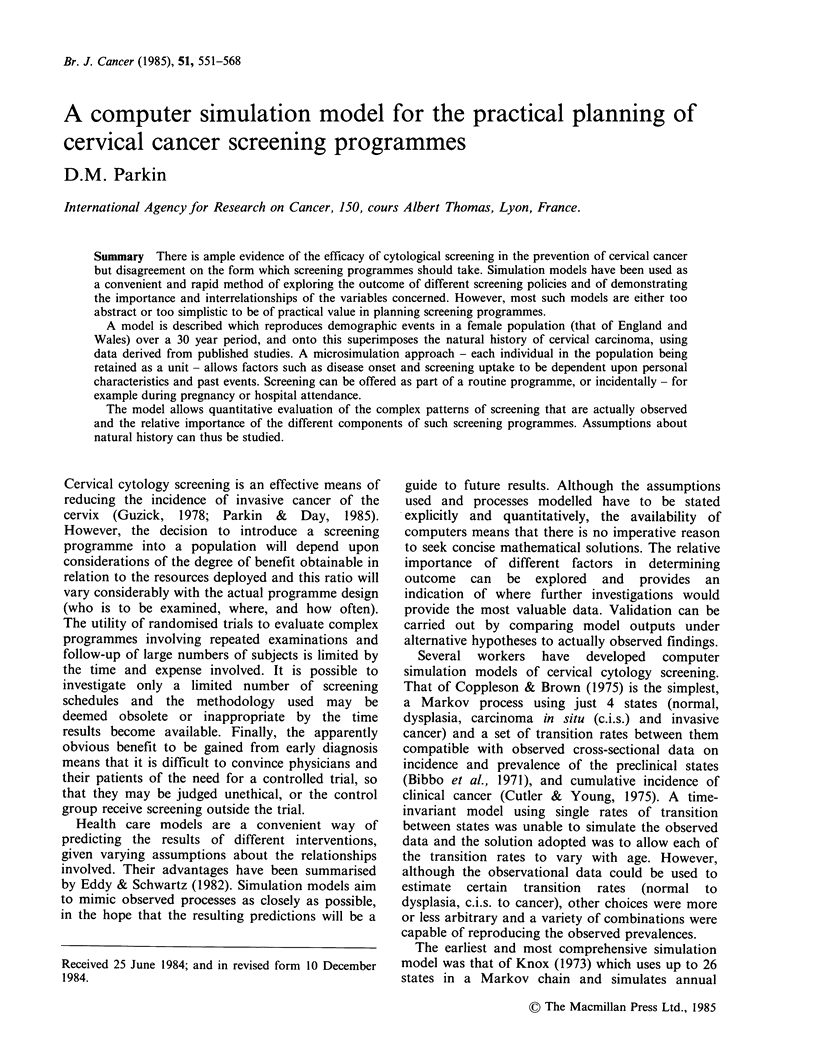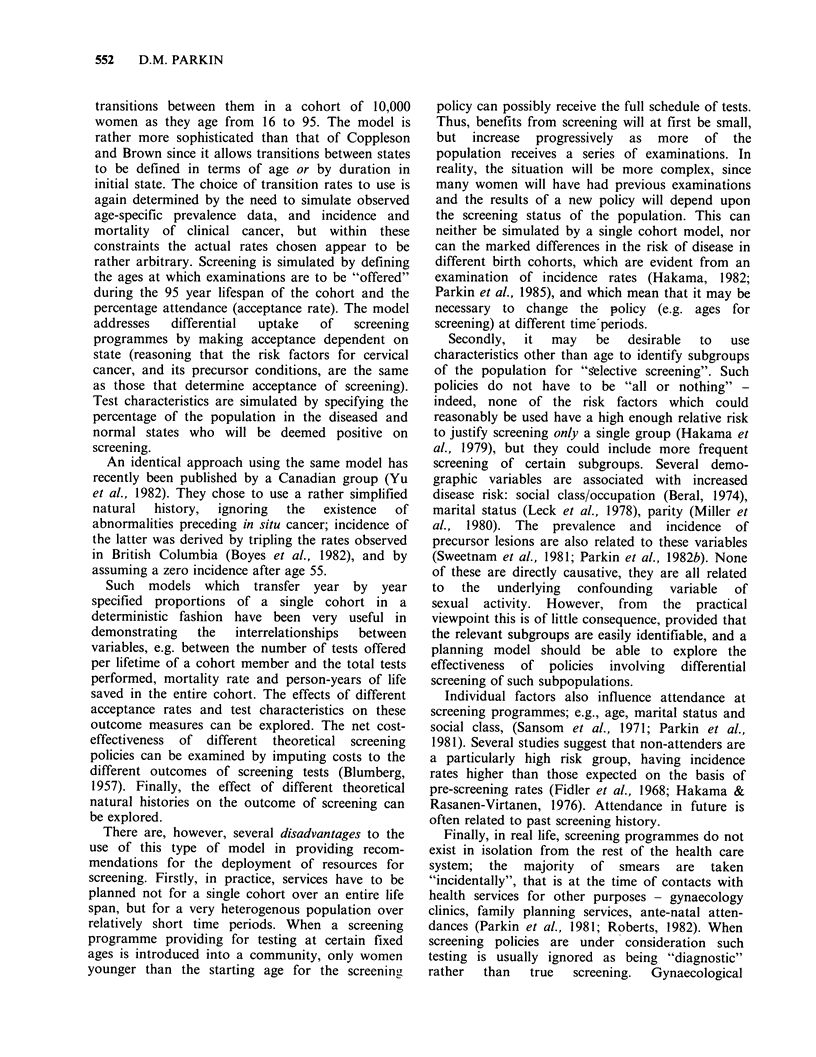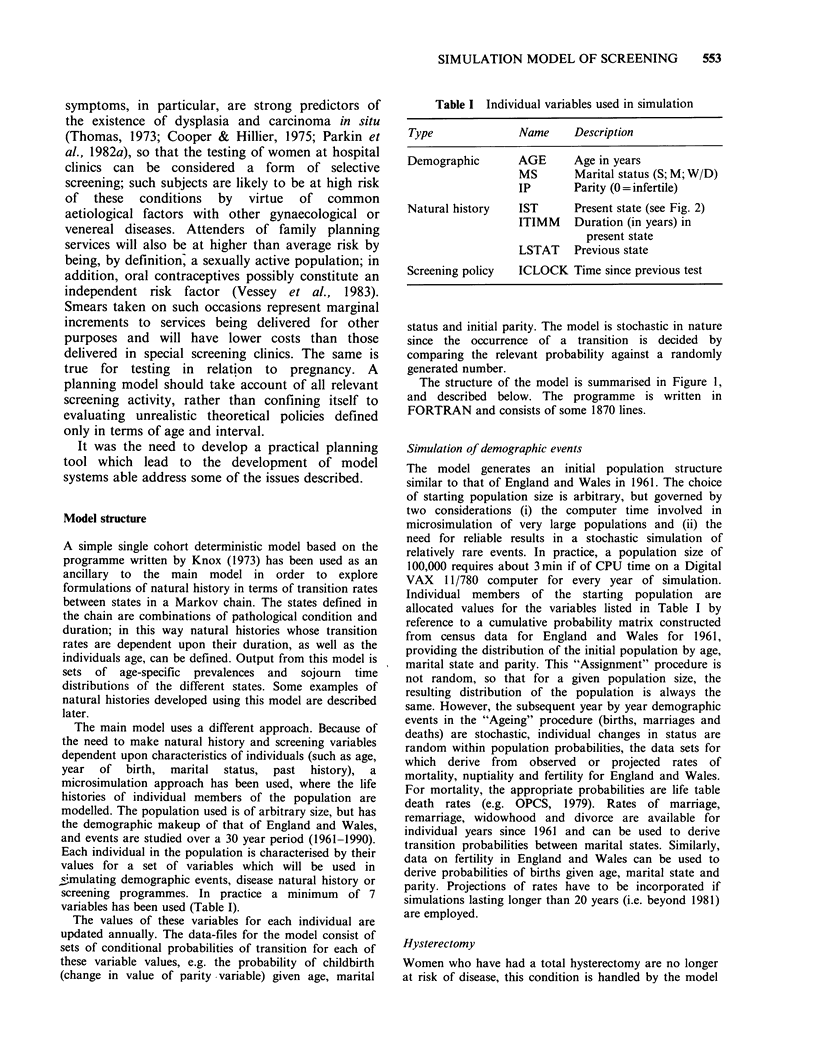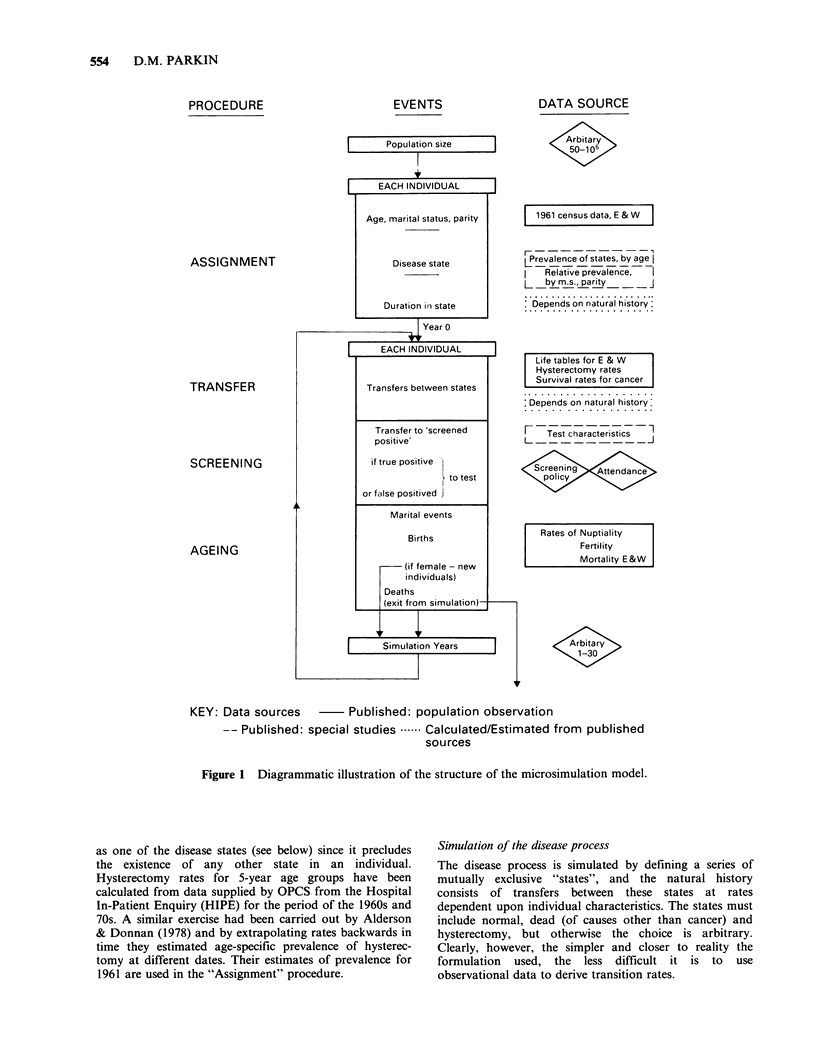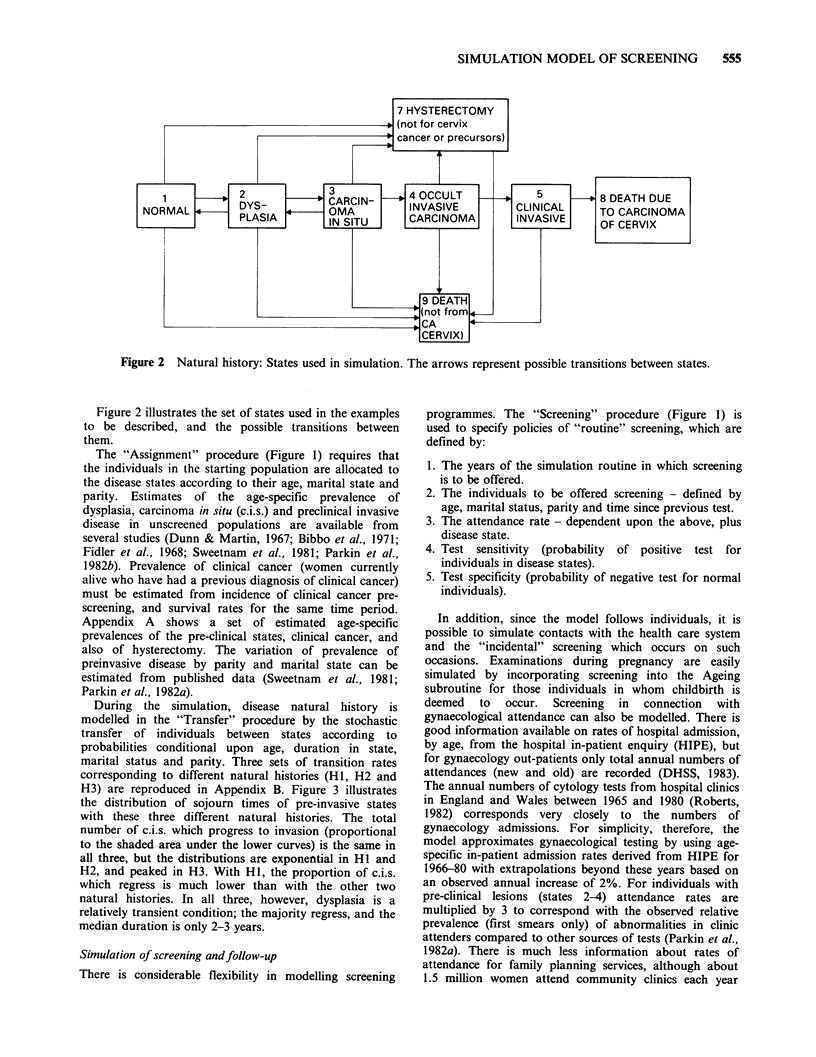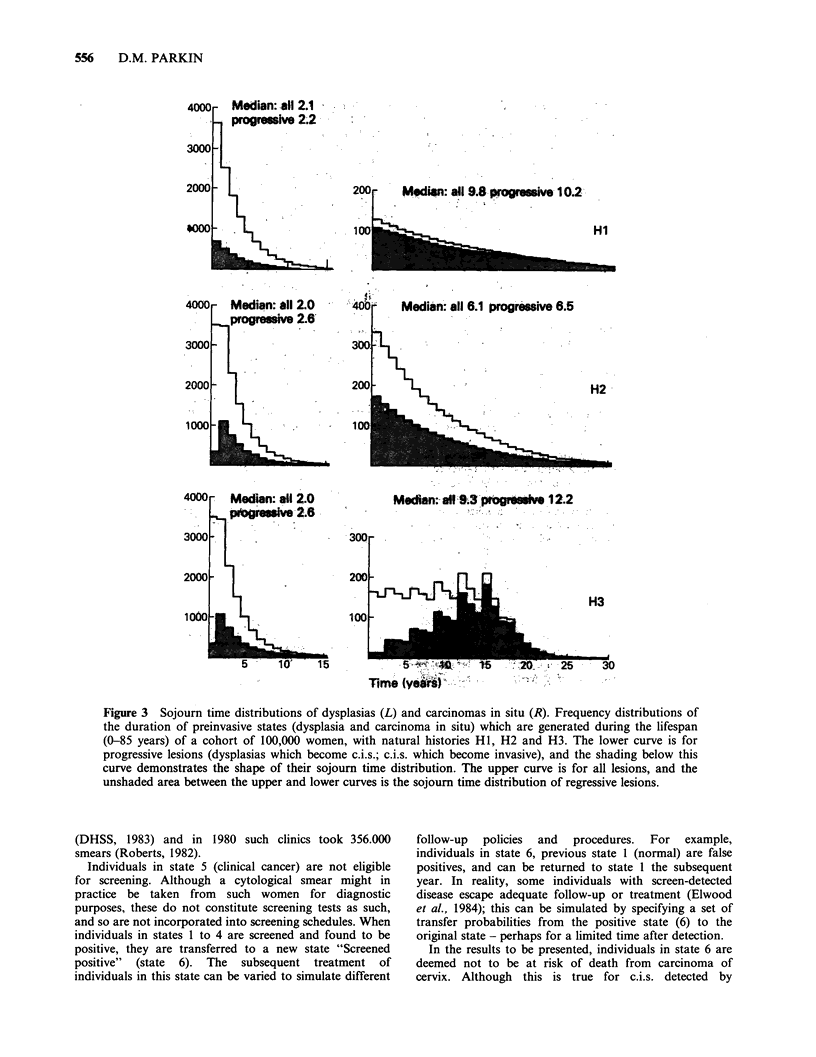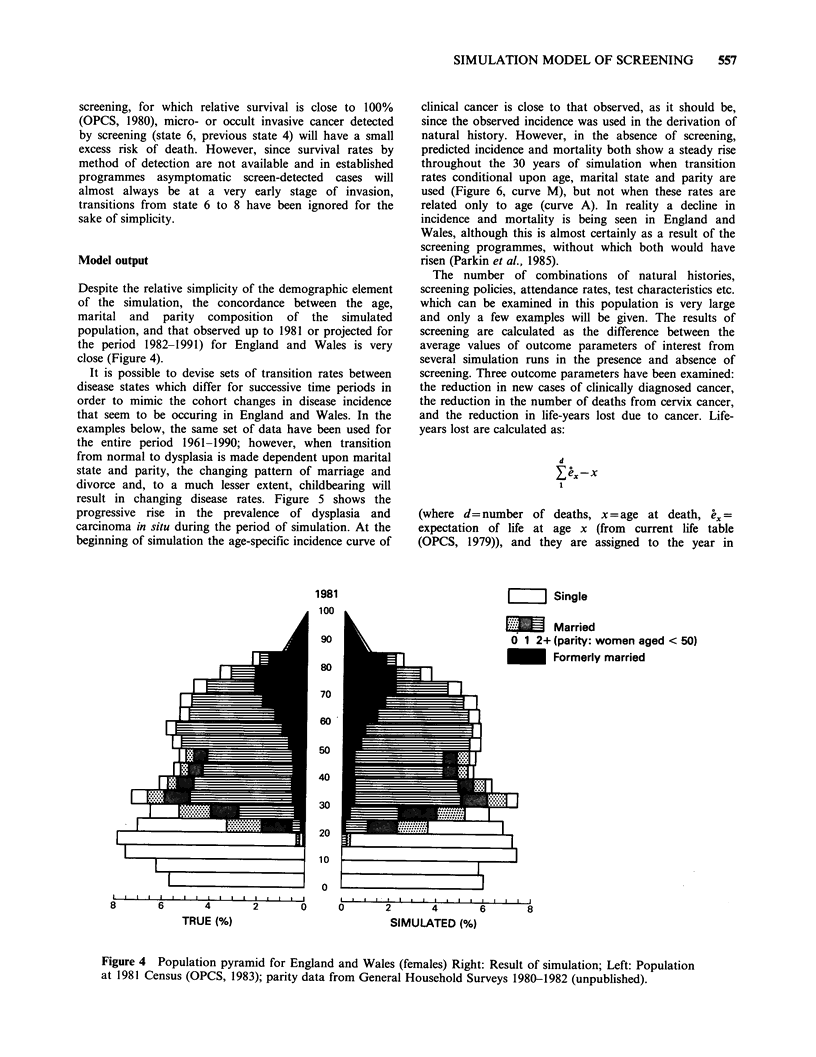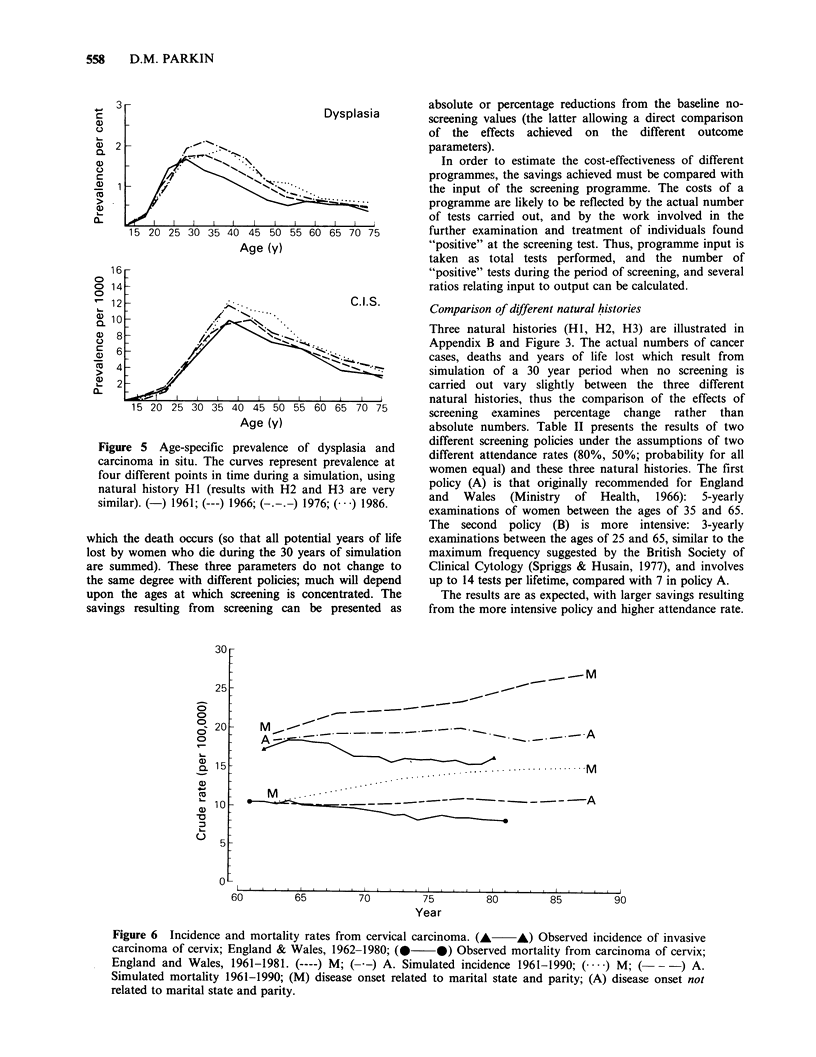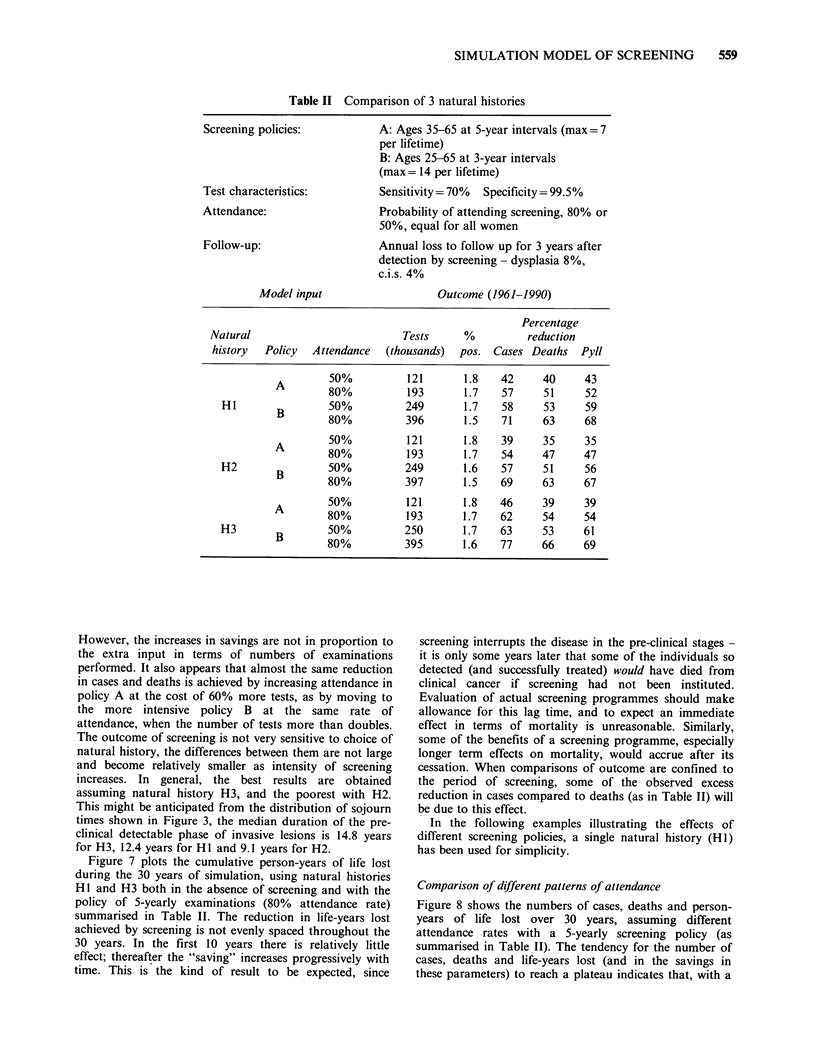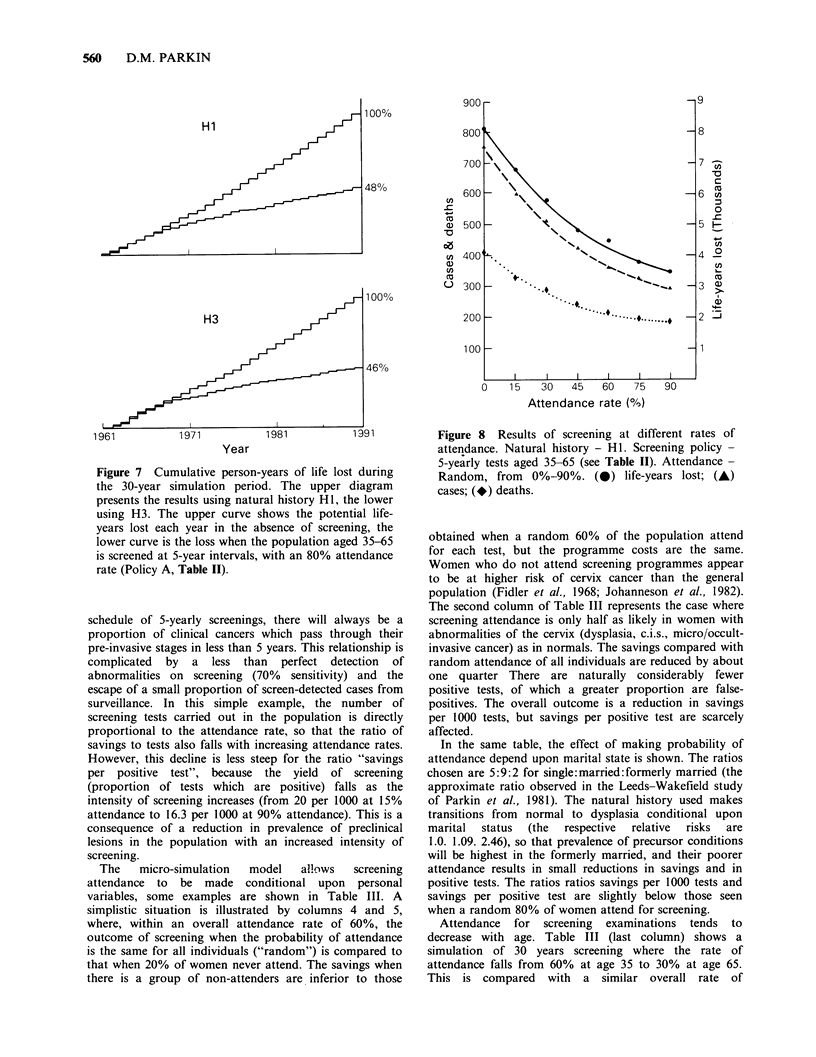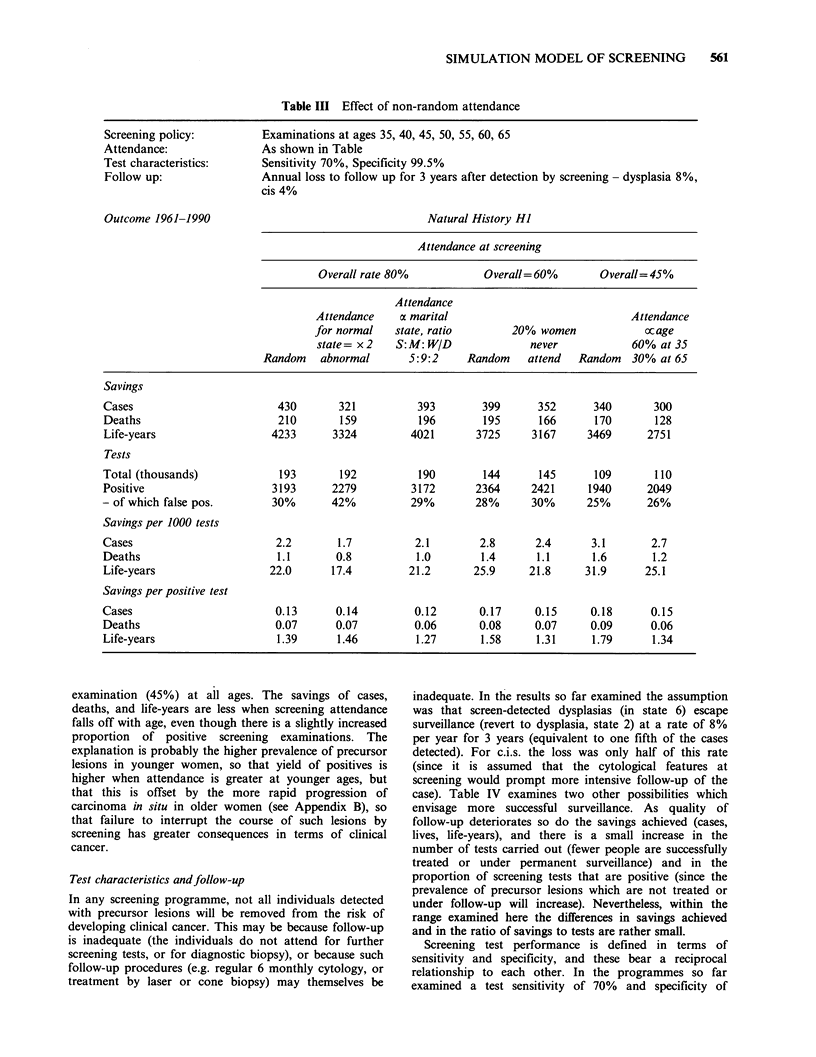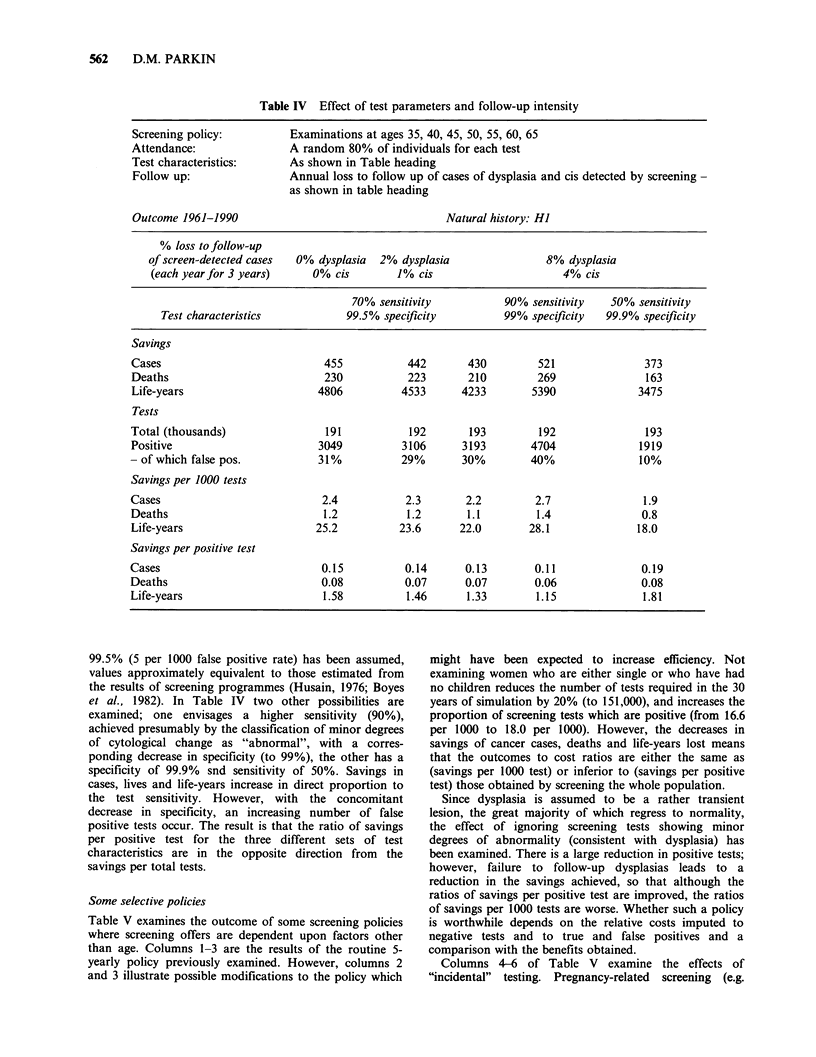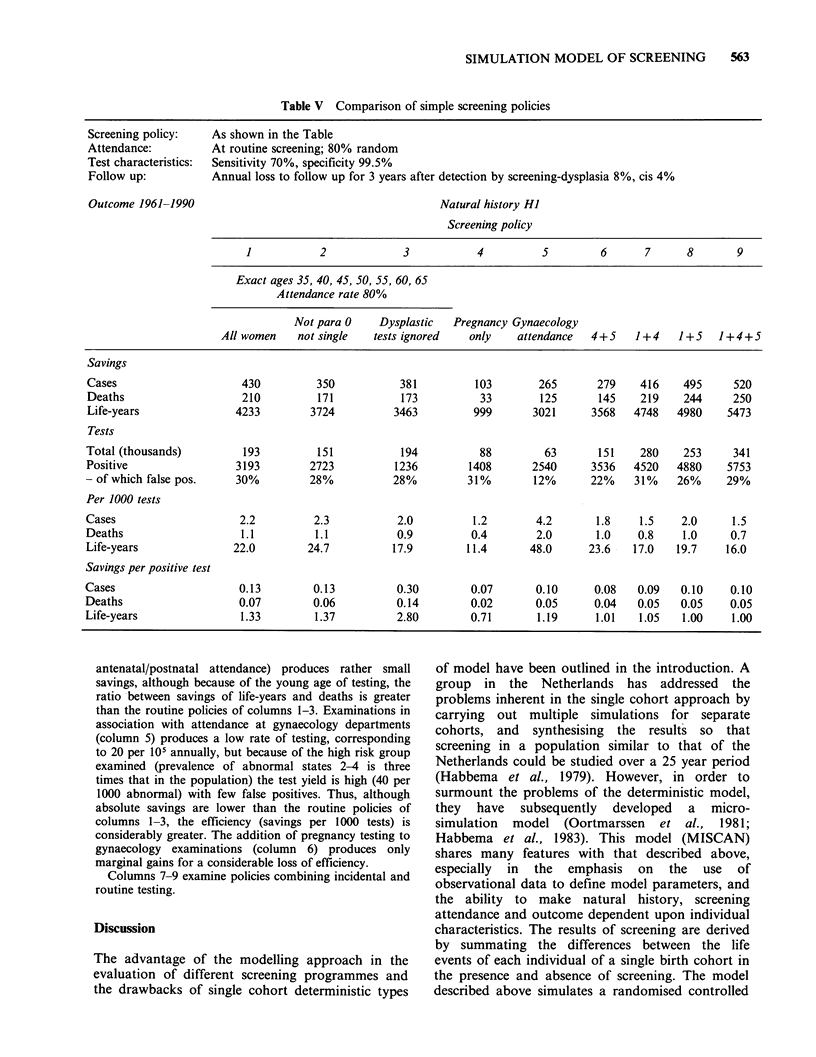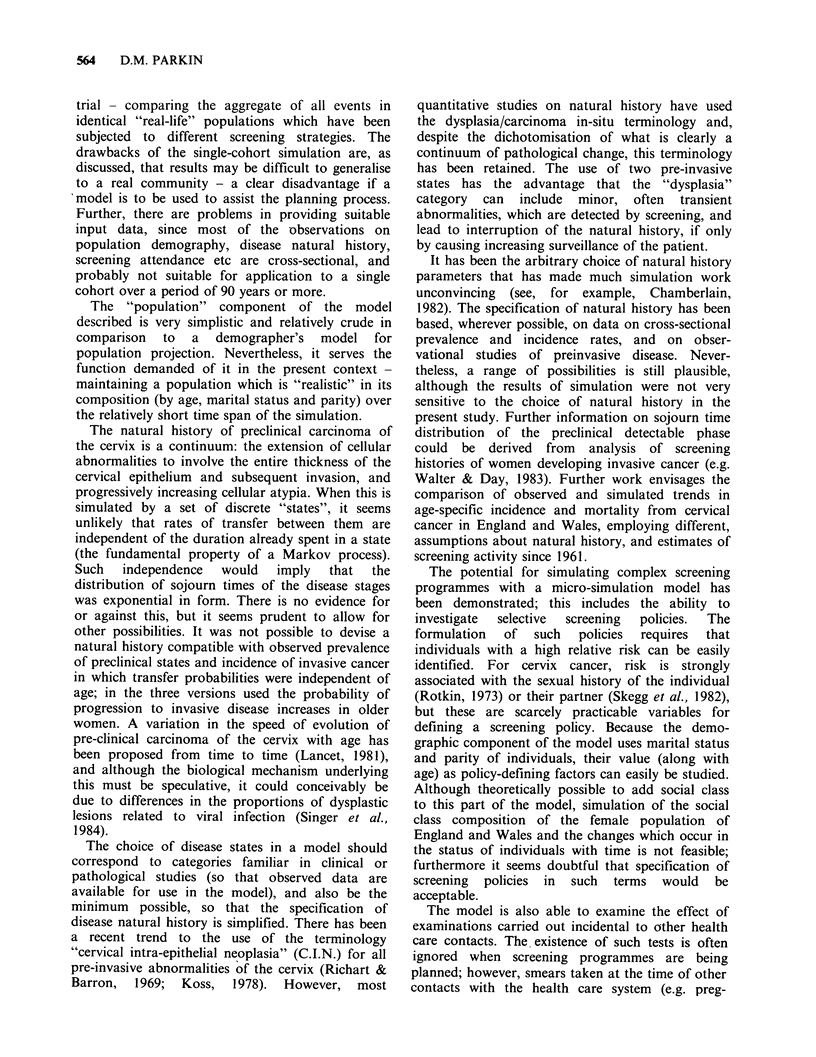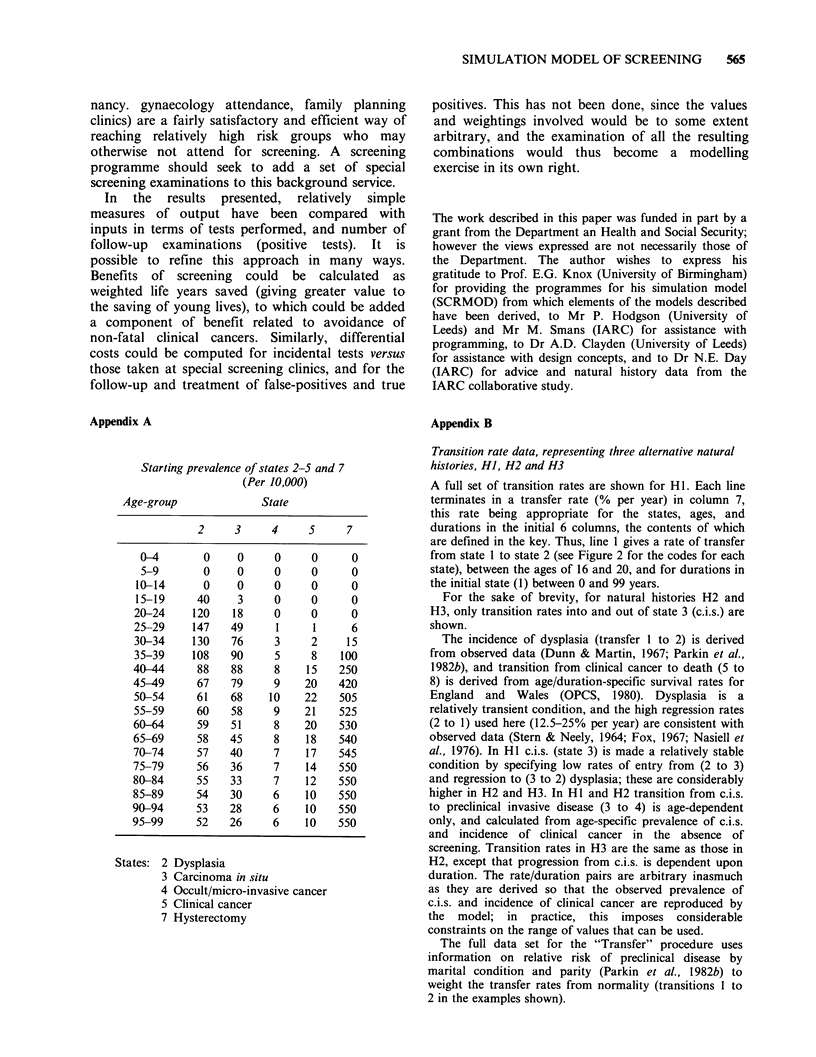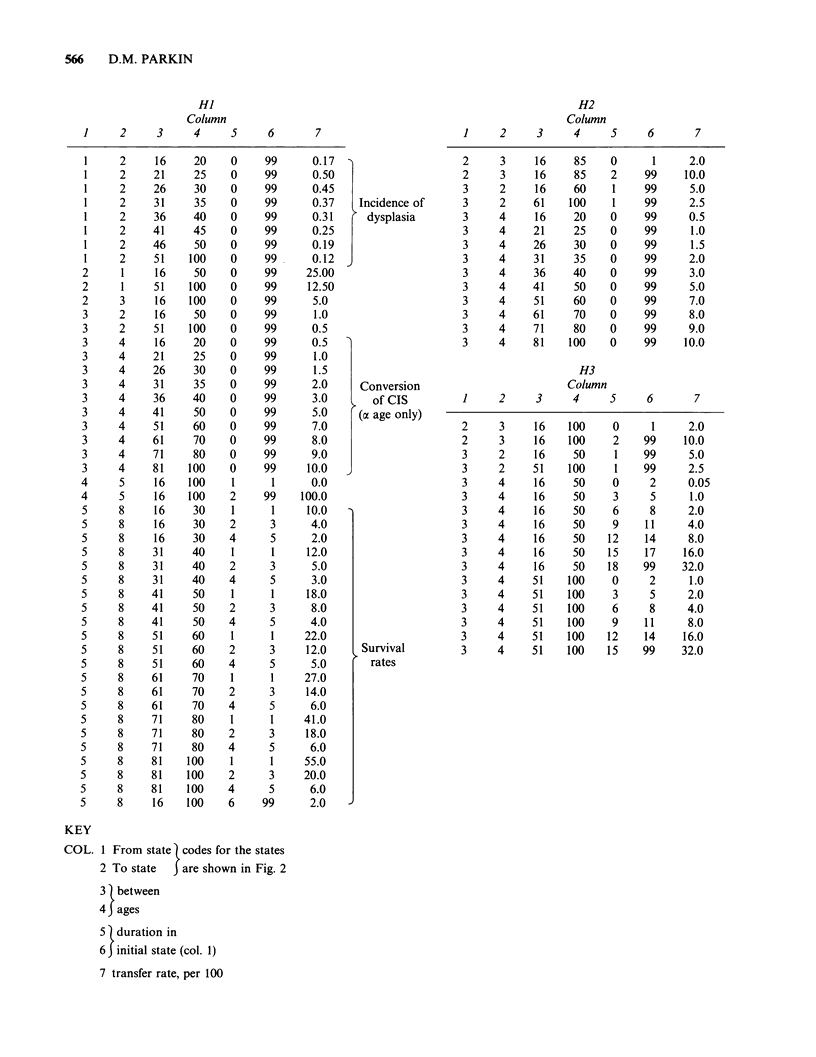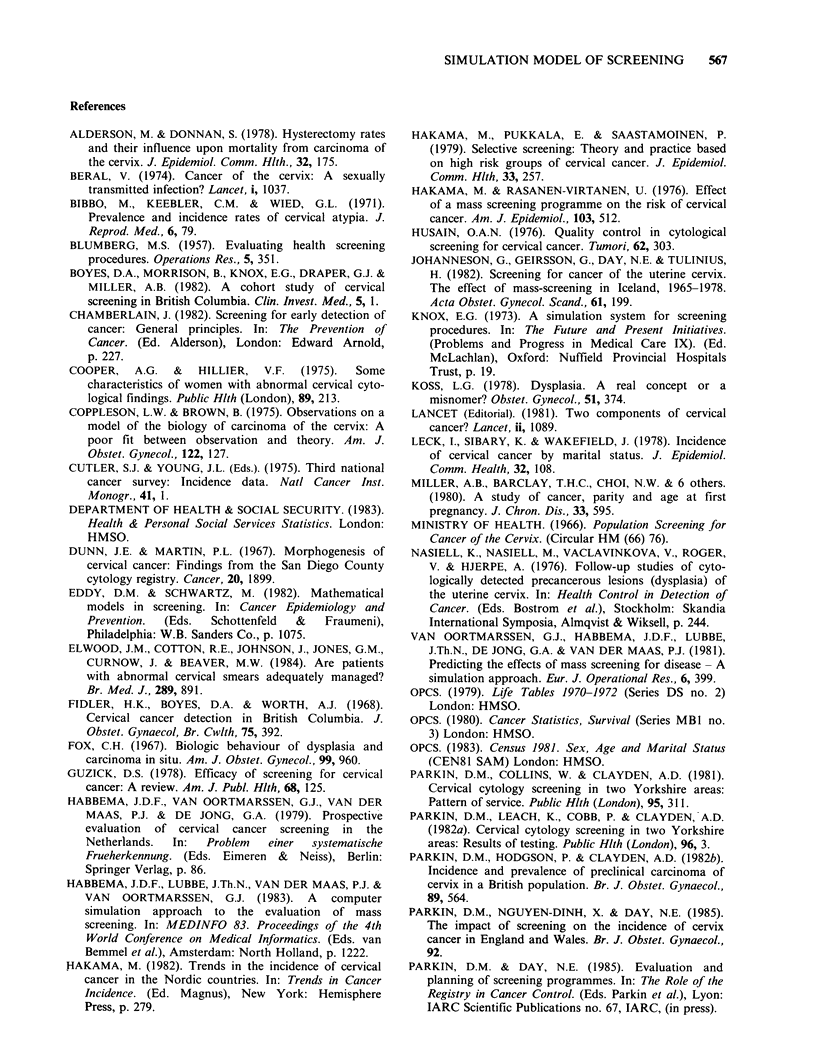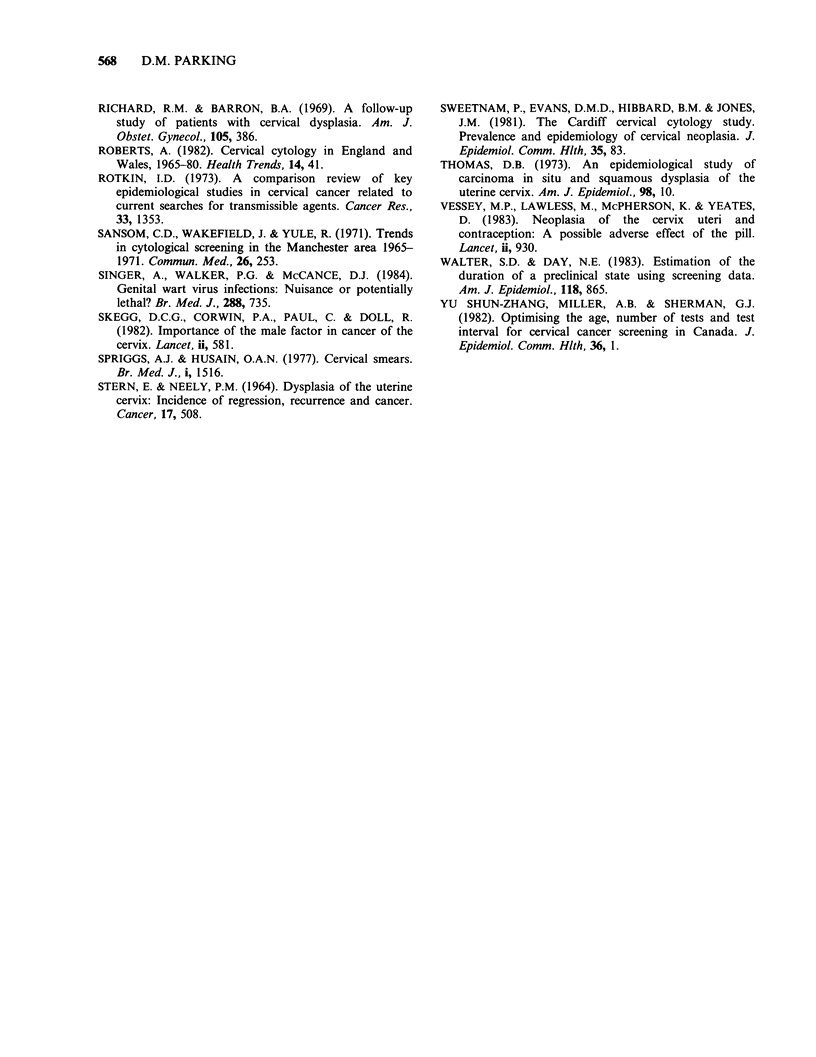# A computer simulation model for the practical planning of cervical cancer screening programmes.

**DOI:** 10.1038/bjc.1985.78

**Published:** 1985-04

**Authors:** D. M. Parkin

## Abstract

**Images:**


*Br. J. Cancer* (1985), **51**, 551–568

### A computer simulation model for the practical planning of cervical cancer screening programmes

D.M. Parkin

*International Agency for Research on Cancer, 150, cours Albert Thomas, Lyon, France.*

**Summary** There is ample evidence of the efficacy of cytological screening in the prevention of cervical cancer but disagreement on the form which screening programmes should take. Simulation models have been used as a convenient and rapid method of exploring the outcome of different screening policies and of demonstrating the importance and interrelationships of the variables concerned. However, most such models are either too abstract or too simplistic to be of practical value in planning screening programmes.

A model is described which reproduces demographic events in a female population (that of England and Wales) over a 30 year period, and onto this superimposes the natural history of cervical carcinoma, using data derived from published studies. A microsimulation approach – each individual in the population being retained as a unit – allows factors such as disease onset and screening uptake to be dependent upon personal characteristics and past events. Screening can be offered as part of a routine programme, or incidentally – for example during pregnancy or hospital attendance.

The model allows quantitative evaluation of the complex patterns of screening that are actually observed and the relative importance of the different components of such screening programmes. Assumptions about natural history can thus be studied.

Cervical cytology screening is an effective means of reducing the incidence of invasive cancer of the cervix (Guzick, 1978; Parkin & Day, 1985). However, the decision to introduce a screening programme into a population will depend upon considerations of the degree of benefit obtainable in relation to the resources deployed and this ratio will vary considerably with the actual programme design (who is to be examined, where, and how often). The utility of randomised trials to evaluate complex programmes involving repeated examinations and follow-up of large numbers of subjects is limited by the time and expense involved. It is possible to investigate only a limited number of screening schedules and the methodology used may be deemed obsolete or inappropriate by the time results become available. Finally, the apparently obvious benefit to be gained from early diagnosis means that it is difficult to convince physicians and their patients of the need for a controlled trial, so that they may be judged unethical, or the control group receive screening outside the trial.

Health care models are a convenient way of predicting the results of different interventions, given varying assumptions about the relationships involved. Their advantages have been summarised by Eddy & Schwartz (1982). Simulation models aim to mimic observed processes as closely as possible, in the hope that the resulting predictions will be a guide to future results. Although the assumptions used and processes modelled have to be stated explicitly and quantitatively, the availability of computers means that there is no imperative reason to seek concise mathematical solutions. The relative importance of different factors in determining outcome can be explored and provides an indication of where further investigations would provide the most valuable data. Validation can be carried out by comparing model outputs under alternative hypotheses to actually observed findings.

Several workers have developed computer simulation models of cervical cytology screening. That of Coppleson & Brown (1975) is the simplest, a Markov process using just 4 states (normal, dysplasia, carcinoma *in situ* (c.i.s.) and invasive cancer) and a set of transition rates between them compatible with observed cross-sectional data on incidence and prevalence of the preclinical states (Bibbo *et al.*, 1971), and cumulative incidence of clinical cancer (Cutler & Young, 1975). A time-invariant model using single rates of transition between states was unable to simulate the observed data and the solution adopted was to allow each of the transition rates to vary with age. However, although the observational data could be used to estimate certain transition rates (normal to dysplasia, c.i.s. to cancer), other choices were more or less arbitrary and a variety of combinations were capable of reproducing the observed prevalences.

The earliest and most comprehensive simulation model was that of Knox (1973) which uses up to 26 states in a Markov chain and simulates annual

Received 25 June 1984; and in revised form 10 December 1984.

© The Macmillan Press Ltd., 1985

transitions between them in a cohort of 10,000 women as they age from 16 to 95. The model is rather more sophisticated than that of Coppleson and Brown since it allows transitions between states to be defined in terms of age *or* by duration in initial state. The choice of transition rates to use is again determined by the need to simulate observed age-specific prevalence data, and incidence and mortality of clinical cancer, but within these constraints the actual rates chosen appear to be rather arbitrary. Screening is simulated by defining the ages at which examinations are to be "offered" during the 95 year lifespan of the cohort and the percentage attendance (acceptance rate). The model addresses differential uptake of screening programmes by making acceptance dependent on state (reasoning that the risk factors for cervical cancer, and its precursor conditions, are the same as those that determine acceptance of screening). Test characteristics are simulated by specifying the percentage of the population in the diseased and normal states who will be deemed positive on screening.

An identical approach using the same model has recently been published by a Canadian group (Yu *et al.*, 1982). They chose to use a rather simplified natural history, ignoring the existence of abnormalities preceding *in situ* cancer; incidence of the latter was derived by tripling the rates observed in British Columbia (Boyes *et al.*, 1982), and by assuming a zero incidence after age 55.

Such models which transfer year by year specified proportions of a single cohort in a deterministic fashion have been very useful in demonstrating the interrelationships between variables, e.g. between the number of tests offered per lifetime of a cohort member and the total tests performed, mortality rate and person-years of life saved in the entire cohort. The effects of different acceptance rates and test characteristics on these outcome measures can be explored. The net cost-effectiveness of different theoretical screening policies can be examined by imputing costs to the different outcomes of screening tests (Blumberg, 1957). Finally, the effect of different theoretical natural histories on the outcome of screening can be explored.

There are, however, several *disadvantages* to the use of this type of model in providing recommendations for the deployment of resources for screening. Firstly, in practice, services have to be planned not for a single cohort over an entire life span, but for a very heterogenous population over relatively short time periods. When a screening programme providing for testing at certain fixed ages is introduced into a community, only women younger than the starting age for the screening policy can possibly receive the full schedule of tests. Thus, benefits from screening will at first be small, but increase progressively as more of the population receives a series of examinations. In reality, the situation will be more complex, since many women will have had previous examinations and the results of a new policy will depend upon the screening status of the population. This can neither be simulated by a single cohort model, nor can the marked differences in the risk of disease in different birth cohorts, which are evident from an examination of incidence rates (Hakama, 1982; Parkin *et al.*, 1985), and which mean that it may be necessary to change the policy (e.g. ages for screening) at different time periods.

Secondly, it may be desirable to use characteristics other than age to identify subgroups of the population for "selective screening". Such policies do not have to be "all or nothing" – indeed, none of the risk factors which could reasonably be used have a high enough relative risk to justify screening *only* a single group (Hakama *et al.*, 1979), but they could include more frequent screening of certain subgroups. Several demographic variables are associated with increased disease risk: social class/occupation (Beral, 1974), marital status (Leck *et al.*, 1978), parity (Miller *et al.*, 1980). The prevalence and incidence of precursor lesions are also related to these variables (Sweetnam *et al.*, 1981; Parkin *et al.*, 1982*b*). None of these are directly causative, they are all related to the underlying confounding variable of sexual activity. However, from the practical viewpoint this is of little consequence, provided that the relevant subgroups are easily identifiable, and a planning model should be able to explore the effectiveness of policies involving differential screening of such subpopulations.

Individual factors also influence attendance at screening programmes; e.g., age, marital status and social class, (Sansom *et al.*, 1971; Parkin *et al.*, 1981). Several studies suggest that non-attenders are a particularly high risk group, having incidence rates higher than those expected on the basis of pre-screening rates (Fidler *et al.*, 1968; Hakama & Rasanen-Virtanen, 1976). Attendance in future is often related to past screening history.

Finally, in real life, screening programmes do not exist in isolation from the rest of the health care system; the majority of smears are taken "incidentally", that is at the time of contacts with health services for other purposes – gynaecology clinics, family planning services, ante-natal attendances (Parkin *et al.*, 1981; Roberts, 1982). When screening policies are under consideration such testing is usually ignored as being "diagnostic" rather than true screening. Gynaecological symptoms, in particular, are strong predictors of the existence of dysplasia and carcinoma *in situ* (Thomas, 1973; Cooper & Hillier, 1975; Parkin *et al.*, 1982*a*), so that the testing of women at hospital clinics can be considered a form of selective screening; such subjects are likely to be at high risk of these conditions by virtue of common aetiological factors with other gynaecological or venereal diseases. Attenders of family planning services will also be at higher than average risk by being, by definition, a sexually active population; in addition, oral contraceptives possibly constitute an independent risk factor (Vessey *et al.*, 1983). Smears taken on such occasions represent marginal increments to services being delivered for other purposes and will have lower costs than those delivered in special screening clinics. The same is true for testing in relation to pregnancy. A planning model should take account of all relevant screening activity, rather than confining itself to evaluating unrealistic theoretical policies defined only in terms of age and interval.

It was the need to develop a practical planning tool which lead to the development of model systems able address some of the issues described.

### Model structure

A simple single cohort deterministic model based on the programme written by Knox (1973) has been used as an ancillary to the main model in order to explore formulations of natural history in terms of transition rates between states in a Markov chain. The states defined in the chain are combinations of pathological condition and duration; in this way natural histories whose transition rates are dependent upon their duration, as well as the individuals age, can be defined. Output from this model is sets of age-specific prevalences and sojourn time distributions of the different states. Some examples of natural histories developed using this model are described later.

The main model uses a different approach. Because of the need to make natural history and screening variables dependent upon characteristics of individuals (such as age, year of birth, marital status, past history), a microsimulation approach has been used, where the life histories of individual members of the population are modelled. The population used is of arbitrary size, but has the demographic makeup of that of England and Wales, and events are studied over a 30 year period (1961–1990). Each individual in the population is characterised by their values for a set of variables which will be used in simulating demographic events, disease natural history or screening programmes. In practice a minimum of 7 variables has been used (Table I).

**Table I** Individual variables used in simulation

| *Type* | *Name* | *Description* |
|---|---|---|
| Demographic | AGE | Age in years |
| | MS | Marital status (S; M; W/D) |
| | IP | Parity (0 = infertile) |
| Natural history | IST | Present state (see Fig. 2) |
| | ITIMM | Duration (in years) in present state |
| | LSTAT | Previous state |
| Screening policy | ICLOCK | Time since previous test |

The values of these variables for each individual are updated annually. The data-files for the model consist of sets of conditional probabilities of transition for each of these variable values, e.g. the probability of childbirth (change in value of parity variable) given age, marital status and initial parity. The model is stochastic in nature since the occurrence of a transition is decided by comparing the relevant probability against a randomly generated number.

The structure of the model is summarised in Figure 1, and described below. The programme is written in FORTRAN and consists of some 1870 lines.

### *Simulation of demographic events*

The model generates an initial population structure similar to that of England and Wales in 1961. The choice of starting population size is arbitrary, but governed by two considerations (i) the computer time involved in microsimulation of very large populations and (ii) the need for reliable results in a stochastic simulation of relatively rare events. In practice, a population size of 100,000 requires about 3 min if of CPU time on a Digital VAX 11/780 computer for every year of simulation. Individual members of the starting population are allocated values for the variables listed in Table I by reference to a cumulative probability matrix constructed from census data for England and Wales for 1961, providing the distribution of the initial population by age, marital state and parity. This "Assignment" procedure is not random, so that for a given population size, the resulting distribution of the population is always the same. However, the subsequent year by year demographic events in the "Ageing" procedure (births, marriages and deaths) are stochastic, individual changes in status are random within population probabilities, the data sets for which derive from observed or projected rates of mortality, nuptiality and fertility for England and Wales. For mortality, the appropriate probabilities are life table death rates (e.g. OPCS, 1979). Rates of marriage, remarriage, widowhood and divorce are available for individual years since 1961 and can be used to derive transition probabilities between marital states. Similarly, data on fertility in England and Wales can be used to derive probabilities of births given age, marital state and parity. Projections of rates have to be incorporated if simulations lasting longer than 20 years (i.e. beyond 1981) are employed.

### *Hysterectomy*

Women who have had a total hysterectomy are no longer at risk of disease, this condition is handled by the model

**Figure 1** Diagrammatic illustration of the structure of the microsimulation model.

as one of the disease states (see below) since it precludes the existence of any other state in an individual. Hysterectomy rates for 5-year age groups have been calculated from data supplied by OPCS from the Hospital In-Patient Enquiry (HIPE) for the period of the 1960s and 70s. A similar exercise had been carried out by Alderson & Donnan (1978) and by extrapolating rates backwards in time they estimated age-specific prevalence of hysterectomy at different dates. Their estimates of prevalence for 1961 are used in the "Assignment" procedure.

*Simulation of the disease process*

The disease process is simulated by defining a series of mutually exclusive "states", and the natural history consists of transfers between these states at rates dependent upon individual characteristics. The states must include normal, dead (of causes other than cancer) and hysterectomy, but otherwise the choice is arbitrary. Clearly, however, the simpler and closer to reality the formulation used, the less difficult it is to use observational data to derive transition rates.

**Figure 2** Natural history: States used in simulation. The arrows represent possible transitions between states.

Figure 2 illustrates the set of states used in the examples to be described, and the possible transitions between them.

The "Assignment" procedure (Figure 1) requires that the individuals in the starting population are allocated to the disease states according to their age, marital state and parity. Estimates of the age-specific prevalence of dysplasia, carcinoma *in situ* (c.i.s.) and preclinical invasive disease in unscreened populations are available from several studies (Dunn & Martin, 1967; Bibbo *et al.*, 1971; Fidler *et al.*, 1968; Sweetnam *et al.*, 1981; Parkin *et al.*, 1982*b*). Prevalence of clinical cancer (women currently alive who have had a previous diagnosis of clinical cancer) must be estimated from incidence of clinical cancer pre-screening, and survival rates for the same time period. Appendix A shows a set of estimated age-specific prevalences of the pre-clinical states, clinical cancer, and also of hysterectomy. The variation of prevalence of preinvasive disease by parity and marital state can be estimated from published data (Sweetnam *et al.*, 1981; Parkin *et al.*, 1982*a*).

During the simulation, disease natural history is modelled in the "Transfer" procedure by the stochastic transfer of individuals between states according to probabilities conditional upon age, duration in state, marital status and parity. Three sets of transition rates corresponding to different natural histories (H1, H2 and H3) are reproduced in Appendix B. Figure 3 illustrates the distribution of sojourn times of pre-invasive states with these three different natural histories. The total number of c.i.s. which progress to invasion (proportional to the shaded area under the lower curves) is the same in all three, but the distributions are exponential in H1 and H2, and peaked in H3. With H1, the proportion of c.i.s. which regress is much lower than with the other two natural histories. In all three, however, dysplasia is a relatively transient condition; the majority regress, and the median duration is only 2–3 years.

*Simulation of screening and follow-up*

There is considerable flexibility in modelling screening programmes. The "Screening" procedure (Figure 1) is used to specify policies of "routine" screening, which are defined by:

1. The years of the simulation routine in which screening is to be offered.
2. The individuals to be offered screening – defined by age, marital status, parity and time since previous test.
3. The attendance rate – dependent upon the above, plus disease state.
4. Test sensitivity (probability of positive test for individuals in disease states).
5. Test specificity (probability of negative test for normal individuals).

In addition, since the model follows individuals, it is possible to simulate contacts with the health care system and the "incidental" screening which occurs on such occasions. Examinations during pregnancy are easily simulated by incorporating screening into the Ageing subroutine for those individuals in whom childbirth is deemed to occur. Screening in connection with gynaecological attendance can also be modelled. There is good information available on rates of hospital admission, by age, from the hospital in-patient enquiry (HIPE), but for gynaecology out-patients only total annual numbers of attendances (new and old) are recorded (DHSS, 1983). The annual numbers of cytology tests from hospital clinics in England and Wales between 1965 and 1980 (Roberts, 1982) corresponds very closely to the numbers of gynaecology admissions. For simplicity, therefore, the model approximates gynaecological testing by using age-specific in-patient admission rates derived from HIPE for 1966–80 with extrapolations beyond these years based on an observed annual increase of 2%. For individuals with pre-clinical lesions (states 2–4) attendance rates are multiplied by 3 to correspond with the observed relative prevalence (first smears only) of abnormalities in clinic attenders compared to other sources of tests (Parkin *et al.*, 1982*a*). There is much less information about rates of attendance for family planning services, although about 1.5 million women attend community clinics each year

**Figure 3** Sojourn time distributions of dysplasias (*L*) and carcinomas in situ (*R*). Frequency distributions of the duration of preinvasive states (dysplasia and carcinoma in situ) which are generated during the lifespan (0–85 years) of a cohort of 100,000 women, with natural histories H1, H2 and H3. The lower curve is for progressive lesions (dysplasias which become c.i.s.; c.i.s. which become invasive), and the shading below this curve demonstrates the shape of their sojourn time distribution. The upper curve is for all lesions, and the unshaded area between the upper and lower curves is the sojourn time distribution of regressive lesions.

(DHSS, 1983) and in 1980 such clinics took 356.000 smears (Roberts, 1982).

Individuals in state 5 (clinical cancer) are not eligible for screening. Although a cytological smear might in practice be taken from such women for diagnostic purposes, these do not constitute screening tests as such, and so are not incorporated into screening schedules. When individuals in states 1 to 4 are screened and found to be positive, they are transferred to a new state "Screened positive" (state 6). The subsequent treatment of individuals in this state can be varied to simulate different follow-up policies and procedures. For example, individuals in state 6, previous state 1 (normal) are false positives, and can be returned to state 1 the subsequent year. In reality, some individuals with screen-detected disease escape adequate follow-up or treatment (Elwood *et al.*, 1984); this can be simulated by specifying a set of transfer probabilities from the positive state (6) to the original state – perhaps for a limited time after detection.

In the results to be presented, individuals in state 6 are deemed not to be at risk of death from carcinoma of cervix. Although this is true for c.i.s. detected by screening, for which relative survival is close to 100% (OPCS, 1980), micro- or occult invasive cancer detected by screening (state 6, previous state 4) will have a small excess risk of death. However, since survival rates by method of detection are not available and in established programmes asymptomatic screen-detected cases will almost always be at a very early stage of invasion, transitions from state 6 to 8 have been ignored for the sake of simplicity.

### Model output

Despite the relative simplicity of the demographic element of the simulation, the concordance between the age, marital and parity composition of the simulated population, and that observed up to 1981 or projected for the period 1982–1991) for England and Wales is very close (Figure 4).

It is possible to devise sets of transition rates between disease states which differ for successive time periods in order to mimic the cohort changes in disease incidence that seem to be occuring in England and Wales. In the examples below, the same set of data have been used for the entire period 1961–1990; however, when transition from normal to dysplasia is made dependent upon marital state and parity, the changing pattern of marriage and divorce and, to a much lesser extent, childbearing will result in changing disease rates. Figure 5 shows the progressive rise in the prevalence of dysplasia and carcinoma *in situ* during the period of simulation. At the beginning of simulation the age-specific incidence curve of clinical cancer is close to that observed, as it should be, since the observed incidence was used in the derivation of natural history. However, in the absence of screening, predicted incidence and mortality both show a steady rise throughout the 30 years of simulation when transition rates conditional upon age, marital state and parity are used (Figure 6, curve M), but not when these rates are related only to age (curve A). In reality a decline in incidence and mortality is being seen in England and Wales, although this is almost certainly as a result of the screening programmes, without which both would have risen (Parkin *et al.*, 1985).

The number of combinations of natural histories, screening policies, attendance rates, test characteristics etc. which can be examined in this population is very large and only a few examples will be given. The results of screening are calculated as the difference between the average values of outcome parameters of interest from several simulation runs in the presence and absence of screening. Three outcome parameters have been examined: the reduction in new cases of clinically diagnosed cancer, the reduction in the number of deaths from cervix cancer, and the reduction in life-years lost due to cancer. Life-years lost are calculated as:

$$\sum_{1}^{d} \mathring{e}_x - x$$

(where $d$=number of deaths, $x$=age at death, $\mathring{e}_x$= expectation of life at age $x$ (from current life table (OPCS, 1979)), and they are assigned to the year in

**Figure 4** Population pyramid for England and Wales (females) Right: Result of simulation; Left: Population at 1981 Census (OPCS, 1983); parity data from General Household Surveys 1980–1982 (unpublished).

**Figure 5** Age-specific prevalence of dysplasia and carcinoma in situ. The curves represent prevalence at four different points in time during a simulation, using natural history H1 (results with H2 and H3 are very similar). (—) 1961; (---) 1966; (-.-.-) 1976; (···) 1986.

which the death occurs (so that all potential years of life lost by women who die during the 30 years of simulation are summed). These three parameters do not change to the same degree with different policies; much will depend upon the ages at which screening is concentrated. The savings resulting from screening can be presented as absolute or percentage reductions from the baseline no-screening values (the latter allowing a direct comparison of the effects achieved on the different outcome parameters).

In order to estimate the cost-effectiveness of different programmes, the savings achieved must be compared with the input of the screening programme. The costs of a programme are likely to be reflected by the actual number of tests carried out, and by the work involved in the further examination and treatment of individuals found "positive" at the screening test. Thus, programme input is taken as total tests performed, and the number of "positive" tests during the period of screening, and several ratios relating input to output can be calculated.

*Comparison of different natural histories*

Three natural histories (H1, H2, H3) are illustrated in Appendix B and Figure 3. The actual numbers of cancer cases, deaths and years of life lost which result from simulation of a 30 year period when no screening is carried out vary slightly between the three different natural histories, thus the comparison of the effects of screening examines percentage change rather than absolute numbers. Table II presents the results of two different screening policies under the assumptions of two different attendance rates (80%, 50%; probability for all women equal) and these three natural histories. The first policy (A) is that originally recommended for England and Wales (Ministry of Health, 1966): 5-yearly examinations of women between the ages of 35 and 65. The second policy (B) is more intensive: 3-yearly examinations between the ages of 25 and 65, similar to the maximum frequency suggested by the British Society of Clinical Cytology (Spriggs & Husain, 1977), and involves up to 14 tests per lifetime, compared with 7 in policy A.

The results are as expected, with larger savings resulting from the more intensive policy and higher attendance rate.

**Figure 6** Incidence and mortality rates from cervical carcinoma. (▲——▲) Observed incidence of invasive carcinoma of cervix; England & Wales, 1962–1980; (●——●) Observed mortality from carcinoma of cervix; England and Wales, 1961–1981. (----) M; (-·-) A. Simulated incidence 1961–1990; (····) M; (— — —) A. Simulated mortality 1961–1990; (M) disease onset related to marital state and parity; (A) disease onset *not* related to marital state and parity.

**Table II** Comparison of 3 natural histories

| | |
|---|---|
| Screening policies: | A: Ages 35–65 at 5-year intervals (max = 7 per lifetime)<br>B: Ages 25–65 at 3-year intervals (max = 14 per lifetime) |
| Test characteristics: | Sensitivity = 70% Specificity = 99.5% |
| Attendance: | Probability of attending screening, 80% or 50%, equal for all women |
| Follow-up: | Annual loss to follow up for 3 years after detection by screening – dysplasia 8%, c.i.s. 4% |

| *Model input* | | | *Outcome (1961–1990)* | | | | |
|---|---|---|---|---|---|---|---|
| *Natural history* | *Policy* | *Attendance* | *Tests (thousands)* | *% pos.* | *Percentage reduction Cases* | *Deaths* | *Pyll* |
| H1 | A | 50% | 121 | 1.8 | 42 | 40 | 43 |
| | | 80% | 193 | 1.7 | 57 | 51 | 52 |
| | B | 50% | 249 | 1.7 | 58 | 53 | 59 |
| | | 80% | 396 | 1.5 | 71 | 63 | 68 |
| H2 | A | 50% | 121 | 1.8 | 39 | 35 | 35 |
| | | 80% | 193 | 1.7 | 54 | 47 | 47 |
| | B | 50% | 249 | 1.6 | 57 | 51 | 56 |
| | | 80% | 397 | 1.5 | 69 | 63 | 67 |
| H3 | A | 50% | 121 | 1.8 | 46 | 39 | 39 |
| | | 80% | 193 | 1.7 | 62 | 54 | 54 |
| | B | 50% | 250 | 1.7 | 63 | 53 | 61 |
| | | 80% | 395 | 1.6 | 77 | 66 | 69 |

However, the increases in savings are not in proportion to the extra input in terms of numbers of examinations performed. It also appears that almost the same reduction in cases and deaths is achieved by increasing attendance in policy A at the cost of 60% more tests, as by moving to the more intensive policy B at the same rate of attendance, when the number of tests more than doubles. The outcome of screening is not very sensitive to choice of natural history, the differences between them are not large and become relatively smaller as intensity of screening increases. In general, the best results are obtained assuming natural history H3, and the poorest with H2. This might be anticipated from the distribution of sojourn times shown in Figure 3, the median duration of the pre-clinical detectable phase of invasive lesions is 14.8 years for H3, 12.4 years for H1 and 9.1 years for H2.

Figure 7 plots the cumulative person-years of life lost during the 30 years of simulation, using natural histories H1 and H3 both in the absence of screening and with the policy of 5-yearly examinations (80% attendance rate) summarised in Table II. The reduction in life-years lost achieved by screening is not evenly spaced throughout the 30 years. In the first 10 years there is relatively little effect; thereafter the "saving" increases progressively with time. This is the kind of result to be expected, since screening interrupts the disease in the pre-clinical stages – it is only some years later that some of the individuals so detected (and successfully treated) *would* have died from clinical cancer if screening had not been instituted. Evaluation of actual screening programmes should make allowance for this lag time, and to expect an immediate effect in terms of mortality is unreasonable. Similarly, some of the benefits of a screening programme, especially longer term effects on mortality, would accrue after its cessation. When comparisons of outcome are confined to the period of screening, some of the observed excess reduction in cases compared to deaths (as in Table II) will be due to this effect.

In the following examples illustrating the effects of different screening policies, a single natural history (H1) has been used for simplicity.

*Comparison of different patterns of attendance*

Figure 8 shows the numbers of cases, deaths and person-years of life lost over 30 years, assuming different attendance rates with a 5-yearly screening policy (as summarised in Table II). The tendency for the number of cases, deaths and life-years lost (and in the savings in these parameters) to reach a plateau indicates that, with a

**Figure 7** Cumulative person-years of life lost during the 30-year simulation period. The upper diagram presents the results using natural history H1, the lower using H3. The upper curve shows the potential life-years lost each year in the absence of screening, the lower curve is the loss when the population aged 35–65 is screened at 5-year intervals, with an 80% attendance rate (Policy A, **Table II**).

**Figure 8** Results of screening at different rates of attendance. Natural history – H1. Screening policy – 5-yearly tests aged 35–65 (see **Table II**). Attendance – Random, from 0%–90%. (●) life-years lost; (▲) cases; (◆) deaths.

schedule of 5-yearly screenings, there will always be a proportion of clinical cancers which pass through their pre-invasive stages in less than 5 years. This relationship is complicated by a less than perfect detection of abnormalities on screening (70% sensitivity) and the escape of a small proportion of screen-detected cases from surveillance. In this simple example, the number of screening tests carried out in the population is directly proportional to the attendance rate, so that the ratio of savings to tests also falls with increasing attendance rates. However, this decline is less steep for the ratio "savings per positive test", because the yield of screening (proportion of tests which are positive) falls as the intensity of screening increases (from 20 per 1000 at 15% attendance to 16.3 per 1000 at 90% attendance). This is a consequence of a reduction in prevalence of preclinical lesions in the population with an increased intensity of screening.

The micro-simulation model allows screening attendance to be made conditional upon personal variables, some examples are shown in Table III. A simplistic situation is illustrated by columns 4 and 5, where, within an overall attendance rate of 60%, the outcome of screening when the probability of attendance is the same for all individuals ("random") is compared to that when 20% of women never attend. The savings when there is a group of non-attenders are inferior to those obtained when a random 60% of the population attend for each test, but the programme costs are the same. Women who do not attend screening programmes appear to be at higher risk of cervix cancer than the general population (Fidler *et al.*, 1968; Johanneson *et al.*, 1982). The second column of Table III represents the case where screening attendance is only half as likely in women with abnormalities of the cervix (dysplasia, c.i.s., micro/occult-invasive cancer) as in normals. The savings compared with random attendance of all individuals are reduced by about one quarter There are naturally considerably fewer positive tests, of which a greater proportion are false-positives. The overall outcome is a reduction in savings per 1000 tests, but savings per positive test are scarcely affected.

In the same table, the effect of making probability of attendance depend upon marital state is shown. The ratios chosen are 5:9:2 for single:married:formerly married (the approximate ratio observed in the Leeds–Wakefield study of Parkin *et al.*, 1981). The natural history used makes transitions from normal to dysplasia conditional upon marital status (the respective relative risks are 1.0. 1.09. 2.46), so that prevalence of precursor conditions will be highest in the formerly married, and their poorer attendance results in small reductions in savings and in positive tests. The ratios ratios savings per 1000 tests and savings per positive test are slightly below those seen when a random 80% of women attend for screening.

Attendance for screening examinations tends to decrease with age. Table III (last column) shows a simulation of 30 years screening where the rate of attendance falls from 60% at age 35 to 30% at age 65. This is compared with a similar overall rate of

**Table III** Effect of non-random attendance

| | |
|---|---|
| Screening policy: | Examinations at ages 35, 40, 45, 50, 55, 60, 65 |
| Attendance: | As shown in Table |
| Test characteristics: | Sensitivity 70%, Specificity 99.5% |
| Follow up: | Annual loss to follow up for 3 years after detection by screening – dysplasia 8%, cis 4% |

| *Outcome 1961–1990* | *Natural History H1* | | | | | | |
|---|---|---|---|---|---|---|---|
| | *Attendance at screening* | | | | | | |
| | *Overall rate 80%* | | | *Overall=60%* | | *Overall=45%* | |
| | *Random* | *Attendance for normal state=×2 abnormal* | *Attendance ∝ marital state, ratio S:M:W/D 5:9:2* | *Random* | *20% women never attend* | *Random* | *Attendance ∝ age 60% at 35 30% at 65* |
| *Savings* | | | | | | | |
| Cases | 430 | 321 | 393 | 399 | 352 | 340 | 300 |
| Deaths | 210 | 159 | 196 | 195 | 166 | 170 | 128 |
| Life-years | 4233 | 3324 | 4021 | 3725 | 3167 | 3469 | 2751 |
| *Tests* | | | | | | | |
| Total (thousands) | 193 | 192 | 190 | 144 | 145 | 109 | 110 |
| Positive | 3193 | 2279 | 3172 | 2364 | 2421 | 1940 | 2049 |
| – of which false pos. | 30% | 42% | 29% | 28% | 30% | 25% | 26% |
| *Savings per 1000 tests* | | | | | | | |
| Cases | 2.2 | 1.7 | 2.1 | 2.8 | 2.4 | 3.1 | 2.7 |
| Deaths | 1.1 | 0.8 | 1.0 | 1.4 | 1.1 | 1.6 | 1.2 |
| Life-years | 22.0 | 17.4 | 21.2 | 25.9 | 21.8 | 31.9 | 25.1 |
| *Savings per positive test* | | | | | | | |
| Cases | 0.13 | 0.14 | 0.12 | 0.17 | 0.15 | 0.18 | 0.15 |
| Deaths | 0.07 | 0.07 | 0.06 | 0.08 | 0.07 | 0.09 | 0.06 |
| Life-years | 1.39 | 1.46 | 1.27 | 1.58 | 1.31 | 1.79 | 1.34 |

examination (45%) at all ages. The savings of cases, deaths, and life-years are less when screening attendance falls off with age, even though there is a slightly increased proportion of positive screening examinations. The explanation is probably the higher prevalence of precursor lesions in younger women, so that yield of positives is higher when attendance is greater at younger ages, but that this is offset by the more rapid progression of carcinoma *in situ* in older women (see Appendix B), so that failure to interrupt the course of such lesions by screening has greater consequences in terms of clinical cancer.

*Test characteristics and follow-up*

In any screening programme, not all individuals detected with precursor lesions will be removed from the risk of developing clinical cancer. This may be because follow-up is inadequate (the individuals do not attend for further screening tests, or for diagnostic biopsy), or because such follow-up procedures (e.g. regular 6 monthly cytology, or treatment by laser or cone biopsy) may themselves be inadequate. In the results so far examined the assumption was that screen-detected dysplasias (in state 6) escape surveillance (revert to dysplasia, state 2) at a rate of 8% per year for 3 years (equivalent to one fifth of the cases detected). For c.i.s. the loss was only half of this rate (since it is assumed that the cytological features at screening would prompt more intensive follow-up of the case). Table IV examines two other possibilities which envisage more successful surveillance. As quality of follow-up deteriorates so do the savings achieved (cases, lives, life-years), and there is a small increase in the number of tests carried out (fewer people are successfully treated or under permanent surveillance) and in the proportion of screening tests that are positive (since the prevalence of precursor lesions which are not treated or under follow-up will increase). Nevertheless, within the range examined here the differences in savings achieved and in the ratio of savings to tests are rather small.

Screening test performance is defined in terms of sensitivity and specificity, and these bear a reciprocal relationship to each other. In the programmes so far examined a test sensitivity of 70% and specificity of

**Table IV** Effect of test parameters and follow-up intensity

| | |
|---|---|
| Screening policy: | Examinations at ages 35, 40, 45, 50, 55, 60, 65 |
| Attendance: | A random 80% of individuals for each test |
| Test characteristics: | As shown in Table heading |
| Follow up: | Annual loss to follow up of cases of dysplasia and cis detected by screening – as shown in table heading |

| *Outcome 1961–1990* | *Natural history: H1* | | | | |
|---|---|---|---|---|---|
| *% loss to follow-up of screen-detected cases (each year for 3 years)* | *0% dysplasia 0% cis* | *2% dysplasia 1% cis* | *8% dysplasia 4% cis* | | |
| *Test characteristics* | *70% sensitivity 99.5% specificity* | | | *90% sensitivity 99% specificity* | *50% sensitivity 99.9% specificity* |
| *Savings* | | | | | |
| Cases | 455 | 442 | 430 | 521 | 373 |
| Deaths | 230 | 223 | 210 | 269 | 163 |
| Life-years | 4806 | 4533 | 4233 | 5390 | 3475 |
| *Tests* | | | | | |
| Total (thousands) | 191 | 192 | 193 | 192 | 193 |
| Positive | 3049 | 3106 | 3193 | 4704 | 1919 |
| – of which false pos. | 31% | 29% | 30% | 40% | 10% |
| *Savings per 1000 tests* | | | | | |
| Cases | 2.4 | 2.3 | 2.2 | 2.7 | 1.9 |
| Deaths | 1.2 | 1.2 | 1.1 | 1.4 | 0.8 |
| Life-years | 25.2 | 23.6 | 22.0 | 28.1 | 18.0 |
| *Savings per positive test* | | | | | |
| Cases | 0.15 | 0.14 | 0.13 | 0.11 | 0.19 |
| Deaths | 0.08 | 0.07 | 0.07 | 0.06 | 0.08 |
| Life-years | 1.58 | 1.46 | 1.33 | 1.15 | 1.81 |

99.5% (5 per 1000 false positive rate) has been assumed, values approximately equivalent to those estimated from the results of screening programmes (Husain, 1976; Boyes *et al.*, 1982). In Table IV two other possibilities are examined; one envisages a higher sensitivity (90%), achieved presumably by the classification of minor degrees of cytological change as "abnormal", with a corresponding decrease in specificity (to 99%), the other has a specificity of 99.9% snd sensitivity of 50%. Savings in cases, lives and life-years increase in direct proportion to the test sensitivity. However, with the concomitant decrease in specificity, an increasing number of false positive tests occur. The result is that the ratio of savings per positive test for the three different sets of test characteristics are in the opposite direction from the savings per total tests.

*Some selective policies*

Table V examines the outcome of some screening policies where screening offers are dependent upon factors other than age. Columns 1–3 are the results of the routine 5-yearly policy previously examined. However, columns 2 and 3 illustrate possible modifications to the policy which might have been expected to increase efficiency. Not examining women who are either single or who have had no children reduces the number of tests required in the 30 years of simulation by 20% (to 151,000), and increases the proportion of screening tests which are positive (from 16.6 per 1000 to 18.0 per 1000). However, the decreases in savings of cancer cases, deaths and life-years lost means that the outcomes to cost ratios are either the same as (savings per 1000 test) or inferior to (savings per positive test) those obtained by screening the whole population.

Since dysplasia is assumed to be a rather transient lesion, the great majority of which regress to normality, the effect of ignoring screening tests showing minor degrees of abnormality (consistent with dysplasia) has been examined. There is a large reduction in positive tests; however, failure to follow-up dysplasias leads to a reduction in the savings achieved, so that although the ratios of savings per positive test are improved, the ratios of savings per 1000 tests are worse. Whether such a policy is worthwhile depends on the relative costs imputed to negative tests and to true and false positives and a comparison with the benefits obtained.

Columns 4–6 of Table V examine the effects of "incidental" testing. Pregnancy-related screening (e.g.

**Table V** Comparison of simple screening policies

| | |
|---|---|
| Screening policy: | As shown in the Table |
| Attendance: | At routine screening; 80% random |
| Test characteristics: | Sensitivity 70%, specificity 99.5% |
| Follow up: | Annual loss to follow up for 3 years after detection by screening-dysplasia 8%, cis 4% |

| *Outcome 1961–1990* | *Natural history H1* | | | | | | | | |
|---|---|---|---|---|---|---|---|---|---|
| | *Screening policy* | | | | | | | | |
| | *1* | *2* | *3* | *4* | *5* | *6* | *7* | *8* | *9* |
| | *Exact ages 35, 40, 45, 50, 55, 60, 65 Attendance rate 80%* | | | | | | | | |
| | *All women* | *Not para 0 not single* | *Dysplastic tests ignored* | *Pregnancy only* | *Gynaecology attendance* | *4+5* | *1+4* | *1+5* | *1+4+5* |
| *Savings* | | | | | | | | | |
| Cases | 430 | 350 | 381 | 103 | 265 | 279 | 416 | 495 | 520 |
| Deaths | 210 | 171 | 173 | 33 | 125 | 145 | 219 | 244 | 250 |
| Life-years | 4233 | 3724 | 3463 | 999 | 3021 | 3568 | 4748 | 4980 | 5473 |
| *Tests* | | | | | | | | | |
| Total (thousands) | 193 | 151 | 194 | 88 | 63 | 151 | 280 | 253 | 341 |
| Positive | 3193 | 2723 | 1236 | 1408 | 2540 | 3536 | 4520 | 4880 | 5753 |
| – of which false pos. | 30% | 28% | 28% | 31% | 12% | 22% | 31% | 26% | 29% |
| *Per 1000 tests* | | | | | | | | | |
| Cases | 2.2 | 2.3 | 2.0 | 1.2 | 4.2 | 1.8 | 1.5 | 2.0 | 1.5 |
| Deaths | 1.1 | 1.1 | 0.9 | 0.4 | 2.0 | 1.0 | 0.8 | 1.0 | 0.7 |
| Life-years | 22.0 | 24.7 | 17.9 | 11.4 | 48.0 | 23.6 | 17.0 | 19.7 | 16.0 |
| *Savings per positive test* | | | | | | | | | |
| Cases | 0.13 | 0.13 | 0.30 | 0.07 | 0.10 | 0.08 | 0.09 | 0.10 | 0.10 |
| Deaths | 0.07 | 0.06 | 0.14 | 0.02 | 0.05 | 0.04 | 0.05 | 0.05 | 0.05 |
| Life-years | 1.33 | 1.37 | 2.80 | 0.71 | 1.19 | 1.01 | 1.05 | 1.00 | 1.00 |

antenatal/postnatal attendance) produces rather small savings, although because of the young age of testing, the ratio between savings of life-years and deaths is greater than the routine policies of columns 1–3. Examinations in association with attendance at gynaecology departments (column 5) produces a low rate of testing, corresponding to 20 per $10^5$ annually, but because of the high risk group examined (prevalence of abnormal states 2–4 is three times that in the population) the test yield is high (40 per 1000 abnormal) with few false positives. Thus, although absolute savings are lower than the routine policies of columns 1–3, the efficiency (savings per 1000 tests) is considerably greater. The addition of pregnancy testing to gynaecology examinations (column 6) produces only marginal gains for a considerable loss of efficiency.

Columns 7–9 examine policies combining incidental and routine testing.

### Discussion

The advantage of the modelling approach in the evaluation of different screening programmes and the drawbacks of single cohort deterministic types of model have been outlined in the introduction. A group in the Netherlands has addressed the problems inherent in the single cohort approach by carrying out multiple simulations for separate cohorts, and synthesising the results so that screening in a population similar to that of the Netherlands could be studied over a 25 year period (Habbema *et al.*, 1979). However, in order to surmount the problems of the deterministic model, they have subsequently developed a micro-simulation model (Oortmarssen *et al.*, 1981; Habbema *et al.*, 1983). This model (MISCAN) shares many features with that described above, especially in the emphasis on the use of observational data to define model parameters, and the ability to make natural history, screening attendance and outcome dependent upon individual characteristics. The results of screening are derived by summating the differences between the life events of each individual of a single birth cohort in the presence and absence of screening. The model described above simulates a randomised controlled trial – comparing the aggregate of all events in identical "real-life" populations which have been subjected to different screening strategies. The drawbacks of the single-cohort simulation are, as discussed, that results may be difficult to generalise to a real community – a clear disadvantage if a model is to be used to assist the planning process. Further, there are problems in providing suitable input data, since most of the observations on population demography, disease natural history, screening attendance etc are cross-sectional, and probably not suitable for application to a single cohort over a period of 90 years or more.

The "population" component of the model described is very simplistic and relatively crude in comparison to a demographer's model for population projection. Nevertheless, it serves the function demanded of it in the present context – maintaining a population which is "realistic" in its composition (by age, marital status and parity) over the relatively short time span of the simulation.

The natural history of preclinical carcinoma of the cervix is a continuum: the extension of cellular abnormalities to involve the entire thickness of the cervical epithelium and subsequent invasion, and progressively increasing cellular atypia. When this is simulated by a set of discrete "states", it seems unlikely that rates of transfer between them are independent of the duration already spent in a state (the fundamental property of a Markov process). Such independence would imply that the distribution of sojourn times of the disease stages was exponential in form. There is no evidence for or against this, but it seems prudent to allow for other possibilities. It was not possible to devise a natural history compatible with observed prevalence of preclinical states and incidence of invasive cancer in which transfer probabilities were independent of age; in the three versions used the probability of progression to invasive disease increases in older women. A variation in the speed of evolution of pre-clinical carcinoma of the cervix with age has been proposed from time to time (Lancet, 1981), and although the biological mechanism underlying this must be speculative, it could conceivably be due to differences in the proportions of dysplastic lesions related to viral infection (Singer *et al.*, 1984).

The choice of disease states in a model should correspond to categories familiar in clinical or pathological studies (so that observed data are available for use in the model), and also be the minimum possible, so that the specification of disease natural history is simplified. There has been a recent trend to the use of the terminology "cervical intra-epithelial neoplasia" (C.I.N.) for all pre-invasive abnormalities of the cervix (Richart & Barron, 1969; Koss, 1978). However, most quantitative studies on natural history have used the dysplasia/carcinoma in-situ terminology and, despite the dichotomisation of what is clearly a continuum of pathological change, this terminology has been retained. The use of two pre-invasive states has the advantage that the "dysplasia" category can include minor, often transient abnormalities, which are detected by screening, and lead to interruption of the natural history, if only by causing increasing surveillance of the patient.

It has been the arbitrary choice of natural history parameters that has made much simulation work unconvincing (see, for example, Chamberlain, 1982). The specification of natural history has been based, wherever possible, on data on cross-sectional prevalence and incidence rates, and on observational studies of preinvasive disease. Nevertheless, a range of possibilities is still plausible, although the results of simulation were not very sensitive to the choice of natural history in the present study. Further information on sojourn time distribution of the preclinical detectable phase could be derived from analysis of screening histories of women developing invasive cancer (e.g. Walter & Day, 1983). Further work envisages the comparison of observed and simulated trends in age-specific incidence and mortality from cervical cancer in England and Wales, employing different, assumptions about natural history, and estimates of screening activity since 1961.

The potential for simulating complex screening programmes with a micro-simulation model has been demonstrated; this includes the ability to investigate selective screening policies. The formulation of such policies requires that individuals with a high relative risk can be easily identified. For cervix cancer, risk is strongly associated with the sexual history of the individual (Rotkin, 1973) or their partner (Skegg *et al.*, 1982), but these are scarcely practicable variables for defining a screening policy. Because the demographic component of the model uses marital status and parity of individuals, their value (along with age) as policy-defining factors can easily be studied. Although theoretically possible to add social class to this part of the model, simulation of the social class composition of the female population of England and Wales and the changes which occur in the status of individuals with time is not feasible; furthermore it seems doubtful that specification of screening policies in such terms would be acceptable.

The model is also able to examine the effect of examinations carried out incidental to other health care contacts. The existence of such tests is often ignored when screening programmes are being planned; however, smears taken at the time of other contacts with the health care system (e.g. pregnancy. gynaecology attendance, family planning clinics) are a fairly satisfactory and efficient way of reaching relatively high risk groups who may otherwise not attend for screening. A screening programme should seek to add a set of special screening examinations to this background service.

In the results presented, relatively simple measures of output have been compared with inputs in terms of tests performed, and number of follow-up examinations (positive tests). It is possible to refine this approach in many ways. Benefits of screening could be calculated as weighted life years saved (giving greater value to the saving of young lives), to which could be added a component of benefit related to avoidance of non-fatal clinical cancers. Similarly, differential costs could be computed for incidental tests *versus* those taken at special screening clinics, and for the follow-up and treatment of false-positives and true positives. This has not been done, since the values and weightings involved would be to some extent arbitrary, and the examination of all the resulting combinations would thus become a modelling exercise in its own right.

The work described in this paper was funded in part by a grant from the Department an Health and Social Security; however the views expressed are not necessarily those of the Department. The author wishes to express his gratitude to Prof. E.G. Knox (University of Birmingham) for providing the programmes for his simulation model (SCRMOD) from which elements of the models described have been derived, to Mr P. Hodgson (University of Leeds) and Mr M. Smans (IARC) for assistance with programming, to Dr A.D. Clayden (University of Leeds) for assistance with design concepts, and to Dr N.E. Day (IARC) for advice and natural history data from the IARC collaborative study.

**Appendix A**

*Starting prevalence of states 2–5 and 7*
*(Per 10,000)*

| *Age-group* | *State* | | | | |
|---|---|---|---|---|---|
| | *2* | *3* | *4* | *5* | *7* |
| 0–4 | 0 | 0 | 0 | 0 | 0 |
| 5–9 | 0 | 0 | 0 | 0 | 0 |
| 10–14 | 0 | 0 | 0 | 0 | 0 |
| 15–19 | 40 | 3 | 0 | 0 | 0 |
| 20–24 | 120 | 18 | 0 | 0 | 0 |
| 25–29 | 147 | 49 | 1 | 1 | 6 |
| 30–34 | 130 | 76 | 3 | 2 | 15 |
| 35–39 | 108 | 90 | 5 | 8 | 100 |
| 40–44 | 88 | 88 | 8 | 15 | 250 |
| 45–49 | 67 | 79 | 9 | 20 | 420 |
| 50–54 | 61 | 68 | 10 | 22 | 505 |
| 55–59 | 60 | 58 | 9 | 21 | 525 |
| 60–64 | 59 | 51 | 8 | 20 | 530 |
| 65–69 | 58 | 45 | 8 | 18 | 540 |
| 70–74 | 57 | 40 | 7 | 17 | 545 |
| 75–79 | 56 | 36 | 7 | 14 | 550 |
| 80–84 | 55 | 33 | 7 | 12 | 550 |
| 85–89 | 54 | 30 | 6 | 10 | 550 |
| 90–94 | 53 | 28 | 6 | 10 | 550 |
| 95–99 | 52 | 26 | 6 | 10 | 550 |

States: 2 Dysplasia
3 Carcinoma *in situ*
4 Occult/micro-invasive cancer
5 Clinical cancer
7 Hysterectomy

**Appendix B**

*Transition rate data, representing three alternative natural histories, H1, H2 and H3*

A full set of transition rates are shown for H1. Each line terminates in a transfer rate (% per year) in column 7, this rate being appropriate for the states, ages, and durations in the initial 6 columns, the contents of which are defined in the key. Thus, line 1 gives a rate of transfer from state 1 to state 2 (see Figure 2 for the codes for each state), between the ages of 16 and 20, and for durations in the initial state (1) between 0 and 99 years.

For the sake of brevity, for natural histories H2 and H3, only transition rates into and out of state 3 (c.i.s.) are shown.

The incidence of dysplasia (transfer 1 to 2) is derived from observed data (Dunn & Martin, 1967; Parkin *et al.*, 1982*b*), and transition from clinical cancer to death (5 to 8) is derived from age/duration-specific survival rates for England and Wales (OPCS, 1980). Dysplasia is a relatively transient condition, and the high regression rates (2 to 1) used here (12.5–25% per year) are consistent with observed data (Stern & Neely, 1964; Fox, 1967; Nasiell *et al.*, 1976). In H1 c.i.s. (state 3) is made a relatively stable condition by specifying low rates of entry from (2 to 3) and regression to (3 to 2) dysplasia; these are considerably higher in H2 and H3. In H1 and H2 transition from c.i.s. to preclinical invasive disease (3 to 4) is age-dependent only, and calculated from age-specific prevalence of c.i.s. and incidence of clinical cancer in the absence of screening. Transition rates in H3 are the same as those in H2, except that progression from c.i.s. is dependent upon duration. The rate/duration pairs are arbitrary inasmuch as they are derived so that the observed prevalence of c.i.s. and incidence of clinical cancer are reproduced by the model; in practice, this imposes considerable constraints on the range of values that can be used.

The full data set for the "Transfer" procedure uses information on relative risk of preclinical disease by marital condition and parity (Parkin *et al.*, 1982*b*) to weight the transfer rates from normality (transitions 1 to 2 in the examples shown).

*H1*

| *1* | *2* | *3* | *4* | *5* | *6* | *7* | |
|---|---|---|---|---|---|---|---|
| 1 | 2 | 16 | 20 | 0 | 99 | 0.17 | Incidence of dysplasia |
| 1 | 2 | 21 | 25 | 0 | 99 | 0.50 | |
| 1 | 2 | 26 | 30 | 0 | 99 | 0.45 | |
| 1 | 2 | 31 | 35 | 0 | 99 | 0.37 | |
| 1 | 2 | 36 | 40 | 0 | 99 | 0.31 | |
| 1 | 2 | 41 | 45 | 0 | 99 | 0.25 | |
| 1 | 2 | 46 | 50 | 0 | 99 | 0.19 | |
| 1 | 2 | 51 | 100 | 0 | 99 | 0.12 | |
| 2 | 1 | 16 | 50 | 0 | 99 | 25.00 | |
| 2 | 1 | 51 | 100 | 0 | 99 | 12.50 | |
| 2 | 3 | 16 | 100 | 0 | 99 | 5.0 | |
| 3 | 2 | 16 | 50 | 0 | 99 | 1.0 | |
| 3 | 2 | 51 | 100 | 0 | 99 | 0.5 | |
| 3 | 4 | 16 | 20 | 0 | 99 | 0.5 | Conversion of CIS (α age only) |
| 3 | 4 | 21 | 25 | 0 | 99 | 1.0 | |
| 3 | 4 | 26 | 30 | 0 | 99 | 1.5 | |
| 3 | 4 | 31 | 35 | 0 | 99 | 2.0 | |
| 3 | 4 | 36 | 40 | 0 | 99 | 3.0 | |
| 3 | 4 | 41 | 50 | 0 | 99 | 5.0 | |
| 3 | 4 | 51 | 60 | 0 | 99 | 7.0 | |
| 3 | 4 | 61 | 70 | 0 | 99 | 8.0 | |
| 3 | 4 | 71 | 80 | 0 | 99 | 9.0 | |
| 3 | 4 | 81 | 100 | 0 | 99 | 10.0 | |
| 4 | 5 | 16 | 100 | 1 | 1 | 0.0 | |
| 4 | 5 | 16 | 100 | 2 | 99 | 100.0 | |
| 5 | 8 | 16 | 30 | 1 | 1 | 10.0 | Survival rates |
| 5 | 8 | 16 | 30 | 2 | 3 | 4.0 | |
| 5 | 8 | 16 | 30 | 4 | 5 | 2.0 | |
| 5 | 8 | 31 | 40 | 1 | 1 | 12.0 | |
| 5 | 8 | 31 | 40 | 2 | 3 | 5.0 | |
| 5 | 8 | 31 | 40 | 4 | 5 | 3.0 | |
| 5 | 8 | 41 | 50 | 1 | 1 | 18.0 | |
| 5 | 8 | 41 | 50 | 2 | 3 | 8.0 | |
| 5 | 8 | 41 | 50 | 4 | 5 | 4.0 | |
| 5 | 8 | 51 | 60 | 1 | 1 | 22.0 | |
| 5 | 8 | 51 | 60 | 2 | 3 | 12.0 | |
| 5 | 8 | 51 | 60 | 4 | 5 | 5.0 | |
| 5 | 8 | 61 | 70 | 1 | 1 | 27.0 | |
| 5 | 8 | 61 | 70 | 2 | 3 | 14.0 | |
| 5 | 8 | 61 | 70 | 4 | 5 | 6.0 | |
| 5 | 8 | 71 | 80 | 1 | 1 | 41.0 | |
| 5 | 8 | 71 | 80 | 2 | 3 | 18.0 | |
| 5 | 8 | 71 | 80 | 4 | 5 | 6.0 | |
| 5 | 8 | 81 | 100 | 1 | 1 | 55.0 | |
| 5 | 8 | 81 | 100 | 2 | 3 | 20.0 | |
| 5 | 8 | 81 | 100 | 4 | 5 | 6.0 | |
| 5 | 8 | 16 | 100 | 6 | 99 | 2.0 | |

*H2*

| *1* | *2* | *3* | *4* | *5* | *6* | *7* |
|---|---|---|---|---|---|---|
| 2 | 3 | 16 | 85 | 0 | 1 | 2.0 |
| 2 | 3 | 16 | 85 | 2 | 99 | 10.0 |
| 3 | 2 | 16 | 60 | 1 | 99 | 5.0 |
| 3 | 2 | 61 | 100 | 1 | 99 | 2.5 |
| 3 | 4 | 16 | 20 | 0 | 99 | 0.5 |
| 3 | 4 | 21 | 25 | 0 | 99 | 1.0 |
| 3 | 4 | 26 | 30 | 0 | 99 | 1.5 |
| 3 | 4 | 31 | 35 | 0 | 99 | 2.0 |
| 3 | 4 | 36 | 40 | 0 | 99 | 3.0 |
| 3 | 4 | 41 | 50 | 0 | 99 | 5.0 |
| 3 | 4 | 51 | 60 | 0 | 99 | 7.0 |
| 3 | 4 | 61 | 70 | 0 | 99 | 8.0 |
| 3 | 4 | 71 | 80 | 0 | 99 | 9.0 |
| 3 | 4 | 81 | 100 | 0 | 99 | 10.0 |

*H3*

| *1* | *2* | *3* | *4* | *5* | *6* | *7* |
|---|---|---|---|---|---|---|
| 2 | 3 | 16 | 100 | 0 | 1 | 2.0 |
| 2 | 3 | 16 | 100 | 2 | 99 | 10.0 |
| 3 | 2 | 16 | 50 | 1 | 99 | 5.0 |
| 3 | 2 | 51 | 100 | 1 | 99 | 2.5 |
| 3 | 4 | 16 | 50 | 0 | 2 | 0.05 |
| 3 | 4 | 16 | 50 | 3 | 5 | 1.0 |
| 3 | 4 | 16 | 50 | 6 | 8 | 2.0 |
| 3 | 4 | 16 | 50 | 9 | 11 | 4.0 |
| 3 | 4 | 16 | 50 | 12 | 14 | 8.0 |
| 3 | 4 | 16 | 50 | 15 | 17 | 16.0 |
| 3 | 4 | 16 | 50 | 18 | 99 | 32.0 |
| 3 | 4 | 51 | 100 | 0 | 2 | 1.0 |
| 3 | 4 | 51 | 100 | 3 | 5 | 2.0 |
| 3 | 4 | 51 | 100 | 6 | 8 | 4.0 |
| 3 | 4 | 51 | 100 | 9 | 11 | 8.0 |
| 3 | 4 | 51 | 100 | 12 | 14 | 16.0 |
| 3 | 4 | 51 | 100 | 15 | 99 | 32.0 |

KEY

COL. 1 From state } codes for the states
2 To state } are shown in Fig. 2

3 } between

4 } ages

5 } duration in

6 } initial state (col. 1)

7 transfer rate, per 100

### References

ALDERSON, M. & DONNAN, S. (1978). Hysterectomy rates and their influence upon mortality from carcinoma of the cervix. *J. Epidemiol. Comm. Hlth.*, **32**, 175.

BERAL, V. (1974). Cancer of the cervix: A sexually transmitted infection? *Lancet*, **i**, 1037.

BIBBO, M., KEEBLER, C.M. & WIED, G.L. (1971). Prevalence and incidence rates of cervical atypia. *J. Reprod. Med.*, **6**, 79.

BLUMBERG, M.S. (1957). Evaluating health screening procedures. *Operations Res.*, **5**, 351.

BOYES, D.A., MORRISON, B., KNOX, E.G., DRAPER, G.J. & MILLER, A.B. (1982). A cohort study of cervical screening in British Columbia. *Clin. Invest. Med.*, **5**, 1.

CHAMBERLAIN, J. (1982). Screening for early detection of cancer: General principles. In: *The Prevention of Cancer*. (Ed. Alderson), London: Edward Arnold, p. 227.

COOPER, A.G. & HILLIER, V.F. (1975). Some characteristics of women with abnormal cervical cytological findings. *Public Hlth* (London), **89**, 213.

COPPLESON, L.W. & BROWN, B. (1975). Observations on a model of the biology of carcinoma of the cervix: A poor fit between observation and theory. *Am. J. Obstet. Gynecol.*, **122**, 127.

CUTLER, S.J. & YOUNG, J.L. (Eds.). (1975). Third national cancer survey: Incidence data. *Natl Cancer Inst. Monogr.*, **41**, 1.

DEPARTMENT OF HEALTH & SOCIAL SECURITY. (1983). *Health & Personal Social Services Statistics*. London: HMSO.

DUNN, J.E. & MARTIN, P.L. (1967). Morphogenesis of cervical cancer: Findings from the San Diego County cytology registry. *Cancer*, **20**, 1899.

EDDY, D.M. & SCHWARTZ, M. (1982). Mathematical models in screening. In: *Cancer Epidemiology and Prevention*. (Eds. Schottenfeld & Fraumeni), Philadelphia: W.B. Sanders Co., p. 1075.

ELWOOD, J.M., COTTON, R.E., JOHNSON, J., JONES, G.M., CURNOW, J. & BEAVER, M.W. (1984). Are patients with abnormal cervical smears adequately managed? *Br. Med. J.*, **289**, 891.

FIDLER, H.K., BOYES, D.A. & WORTH, A.J. (1968). Cervical cancer detection in British Columbia. *J. Obstet. Gynaecol, Br. Cwlth*, **75**, 392.

FOX, C.H. (1967). Biologic behaviour of dysplasia and carcinoma in situ. *Am. J. Obstet. Gynecol.*, **99**, 960.

GUZICK, D.S. (1978). Efficacy of screening for cervical cancer: A review. *Am. J. Publ. Hlth*, **68**, 125.

HABBEMA, J.D.F., VAN OORTMARSSEN, G.J., VAN DER MAAS, P.J. & DE JONG, G.A. (1979). Prospective evaluation of cervical cancer screening in the Netherlands. In: *Problem einer systematische Frueherkennung*. (Eds. Eimeren & Neiss), Berlin: Springer Verlag, p. 86.

HABBEMA, J.D.F., LUBBE, J.Th.N., VAN DER MAAS, P.J. & VAN OORTMARSSEN, G.J. (1983). A computer simulation approach to the evaluation of mass screening. In: *MEDINFO 83. Proceedings of the 4th World Conference on Medical Informatics*. (Eds. van Bemmel *et al.*), Amsterdam: North Holland, p. 1222.

HAKAMA, M. (1982). Trends in the incidence of cervical cancer in the Nordic countries. In: *Trends in Cancer Incidence*. (Ed. Magnus), New York: Hemisphere Press, p. 279.

HAKAMA, M., PUKKALA, E. & SAASTAMOINEN, P. (1979). Selective screening: Theory and practice based on high risk groups of cervical cancer. *J. Epidemiol. Comm. Hlth*, **33**, 257.

HAKAMA, M. & RASANEN-VIRTANEN, U. (1976). Effect of a mass screening programme on the risk of cervical cancer. *Am. J. Epidemiol.*, **103**, 512.

HUSAIN, O.A.N. (1976). Quality control in cytological screening for cervical cancer. *Tumori*, **62**, 303.

JOHANNESON, G., GEIRSSON, G., DAY, N.E. & TULINIUS, H. (1982). Screening for cancer of the uterine cervix. The effect of mass-screening in Iceland, 1965–1978. *Acta Obstet. Gynecol. Scand.*, **61**, 199.

KNOX, E.G. (1973). A simulation system for screening procedures. In: *The Future and Present Initiatives*. (Problems and Progress in Medical Care IX). (Ed. McLachlan), Oxford: Nuffield Provincial Hospitals Trust, p. 19.

KOSS, L.G. (1978). Dysplasia. A real concept or a misnomer? *Obstet. Gynecol.*, **51**, 374.

LANCET (Editorial). (1981). Two components of cervical cancer? *Lancet*, **ii**, 1089.

LECK, I., SIBARY, K. & WAKEFIELD, J. (1978). Incidence of cervical cancer by marital status. *J. Epidemiol. Comm. Health*, **32**, 108.

MILLER, A.B., BARCLAY, T.H.C., CHOI, N.W. & 6 others. (1980). A study of cancer, parity and age at first pregnancy. *J. Chron. Dis.*, **33**, 595.

MINISTRY OF HEALTH. (1966). *Population Screening for Cancer of the Cervix*. (Circular HM (66) 76).

NASIELL, K., NASIELL, M., VACLAVINKOVA, V., ROGER, V. & HJERPE, A. (1976). Follow-up studies of cytologically detected precancerous lesions (dysplasia) of the uterine cervix. In: *Health Control in Detection of Cancer*. (Eds. Bostrom *et al.*), Stockholm: Skandia International Symposia, Almqvist & Wiksell, p. 244.

VAN OORTMARSSEN, G.J., HABBEMA, J.D.F., LUBBE, J.Th.N., DE JONG, G.A. & VAN DER MAAS, P.J. (1981). Predicting the effects of mass screening for disease – A simulation approach. *Eur. J. Operational Res.*, **6**, 399.

OPCS. (1979). *Life Tables 1970–1972* (Series DS no. 2) London: HMSO.

OPCS. (1980). *Cancer Statistics, Survival* (Series MB1 no. 3) London: HMSO.

OPCS. (1983). *Census 1981. Sex, Age and Marital Status* (CEN81 SAM) London: HMSO.

PARKIN, D.M., COLLINS, W. & CLAYDEN, A.D. (1981). Cervical cytology screening in two Yorkshire areas: Pattern of service. *Public Hlth* (*London*), **95**, 311.

PARKIN, D.M., LEACH, K., COBB, P. & CLAYDEN, A.D. (1982*a*). Cervical cytology screening in two Yorkshire areas: Results of testing. *Public Hlth* (*London*), **96**, 3.

PARKIN, D.M., HODGSON, P. & CLAYDEN, A.D. (1982*b*). Incidence and prevalence of preclinical carcinoma of cervix in a British population. *Br. J. Obstet. Gynaecol.*, **89**, 564.

PARKIN, D.M., NGUYEN-DINH, X. & DAY, N.E. (1985). The impact of screening on the incidence of cervix cancer in England and Wales. *Br. J. Obstet. Gynaecol.*, **92**.

PARKIN, D.M. & DAY, N.E. (1985). Evaluation and planning of screening programmes. In: *The Role of the Registry in Cancer Control*. (Eds. Parkin *et al.*), Lyon: IARC Scientific Publications no. 67, IARC, (in press).

RICHARD, R.M. & BARRON, B.A. (1969). A follow-up study of patients with cervical dysplasia. *Am. J. Obstet. Gynecol.*, **105**, 386.

ROBERTS, A. (1982). Cervical cytology in England and Wales, 1965–80. *Health Trends*, **14**, 41.

ROTKIN, I.D. (1973). A comparison review of key epidemiological studies in cervical cancer related to current searches for transmissible agents. *Cancer Res.*, **33**, 1353.

SANSOM, C.D., WAKEFIELD, J. & YULE, R. (1971). Trends in cytological screening in the Manchester area 1965–1971. *Commun. Med.*, **26**, 253.

SINGER, A., WALKER, P.G. & McCANCE, D.J. (1984). Genital wart virus infections: Nuisance or potentially lethal? *Br. Med. J.*, **288**, 735.

SKEGG, D.C.G., CORWIN, P.A., PAUL, C. & DOLL, R. (1982). Importance of the male factor in cancer of the cervix. *Lancet*, **ii**, 581.

SPRIGGS, A.J. & HUSAIN, O.A.N. (1977). Cervical smears. *Br. Med. J.*, **i**, 1516.

STERN, E. & NEELY, P.M. (1964). Dysplasia of the uterine cervix: Incidence of regression, recurrence and cancer. *Cancer*, **17**, 508.

SWEETNAM, P., EVANS, D.M.D., HIBBARD, B.M. & JONES, J.M. (1981). The Cardiff cervical cytology study. Prevalence and epidemiology of cervical neoplasia. *J. Epidemiol. Comm. Hlth*, **35**, 83.

THOMAS, D.B. (1973). An epidemiological study of carcinoma in situ and squamous dysplasia of the uterine cervix. *Am. J. Epidemiol.*, **98**, 10.

VESSEY, M.P., LAWLESS, M., McPHERSON, K. & YEATES, D. (1983). Neoplasia of the cervix uteri and contraception: A possible adverse effect of the pill. *Lancet*, **ii**, 930.

WALTER, S.D. & DAY, N.E. (1983). Estimation of the duration of a preclinical state using screening data. *Am. J. Epidemiol.*, **118**, 865.

YU SHUN-ZHANG, MILLER, A.B. & SHERMAN, G.J. (1982). Optimising the age, number of tests and test interval for cervical cancer screening in Canada. *J. Epidemiol. Comm. Hlth*, **36**, 1.